# Supplementary material for: Climate policy implications of nonlinear decline of Arctic land permafrost and other cryosphere elements
Source: Nat Commun. 2019 Apr 23;10:1900. doi: 10.1038/s41467-019-09863-x (PMC6478735; doi:10.1038/s41467-019-09863-x)
Supplement: Supplementary file 1 — Supplementary Information [file 41467_2019_9863_MOESM1_ESM.pdf]

Climate policy implications of nonlinear decline of Arctic land permafrost and other cryosphere elements

Supplementary Information

Yumashev et al., Nature Communications

Supplementary Figure 1

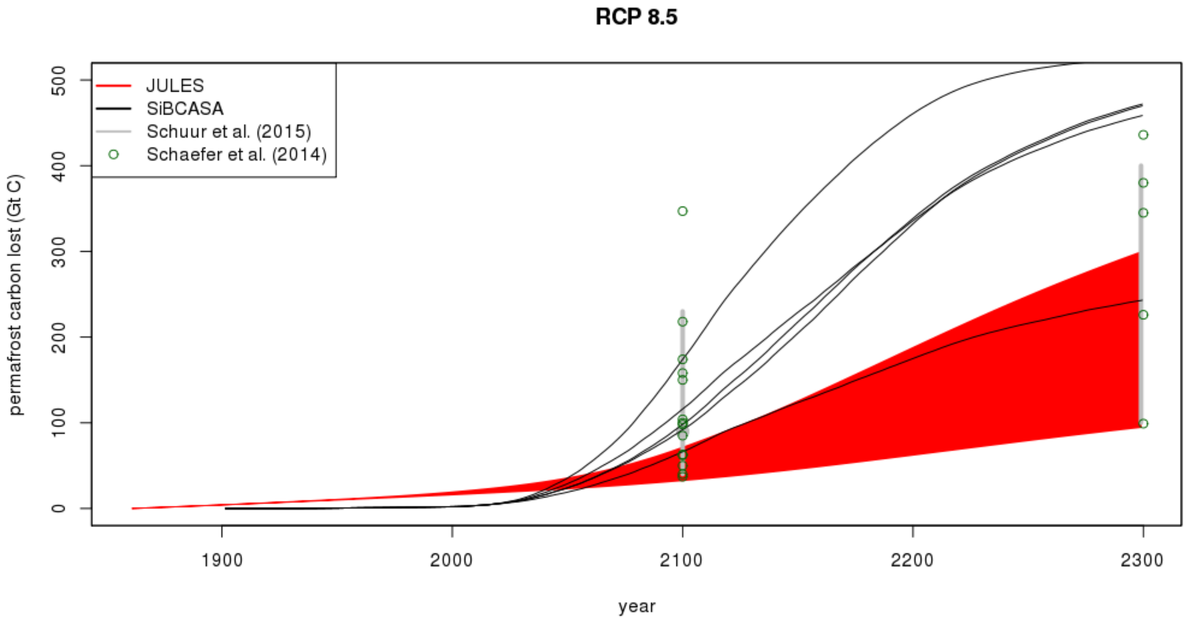

Cumulative permafrost carbon (CO_2 component) for the SiBCASA and JULES simulations with multiple GCMs under RCP8.5. The grey vertical lines are an indication of the permafrost carbon emissions presented in Figure 3 of Schuur et al. (2015). The green circles represent the permafrost carbon emissions reviewed by Schaefer et al. (2014). The spread of values for SiBCASA and JULES encompasses the range of previously published values. Source: Supplementary Data 2, Supplementary Data 3.

Supplementary Figure 2

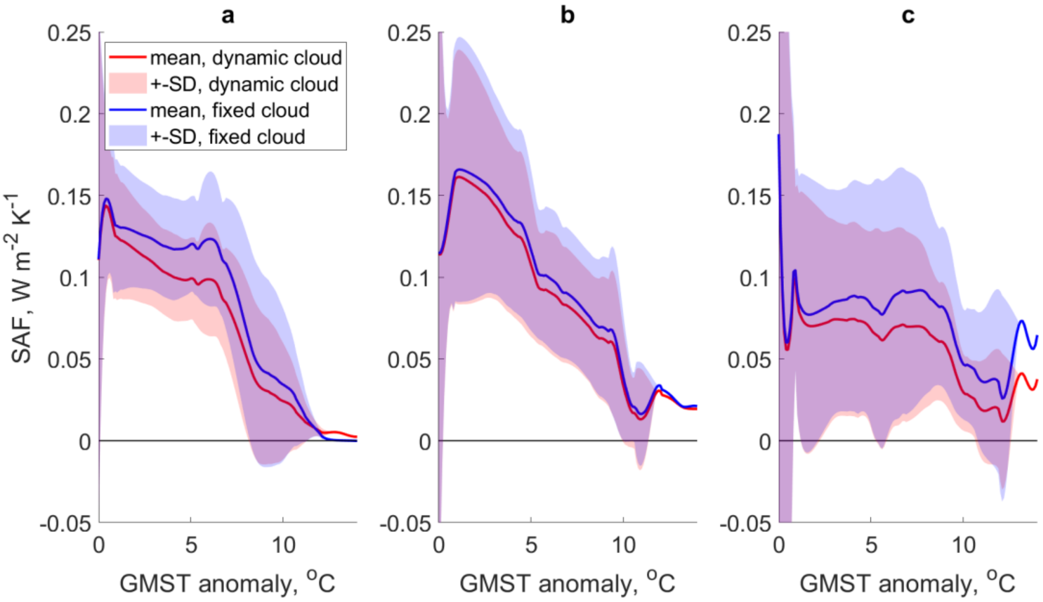

SAF components for (a) Arctic sea ice, (b) land snow and (c) rest of the world expressed as functions of the GMST rise relative to the 1850-1900 pre-industrial conditions. Obtained from multiple CMIP5 GCMs using Winton's ALL/CLR method, assuming dynamic (red) and fixed pre-industrial (blue) cloud covers. Lines: multi-model mean; shaded areas: ±1 SD. Source: Supplementary Code 9.

Supplementary Figure 3

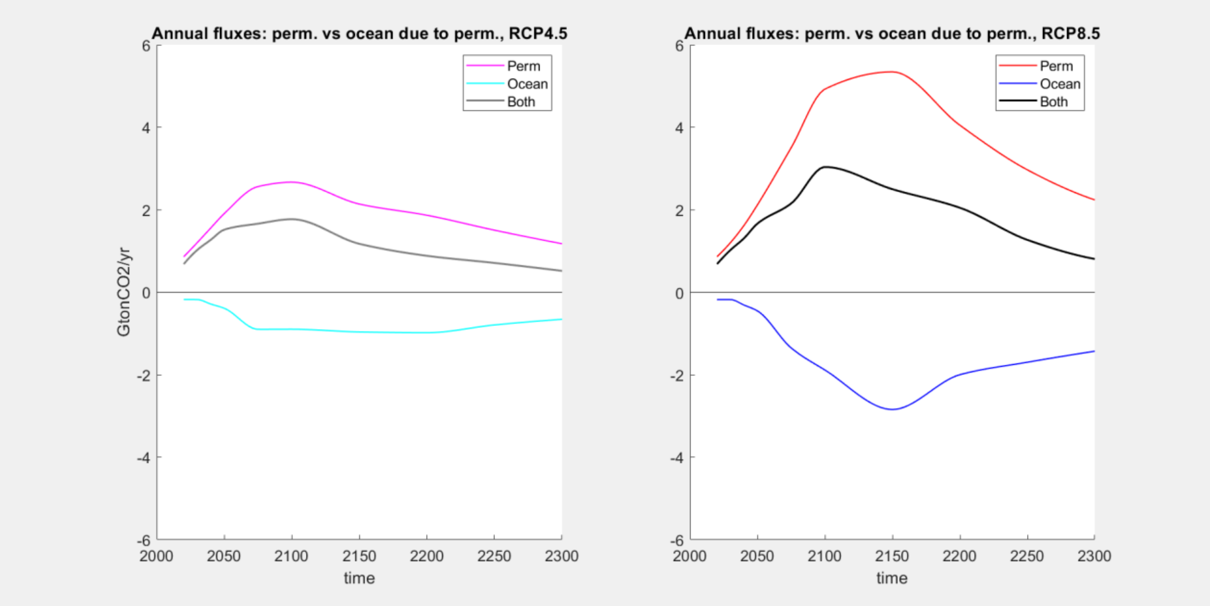

Annual permafrost CO_2 flux after the initial land uptake, and the corresponding annual CO_2 ocean uptake flux driven by the permafrost emissions, as well as the sum of the two, plotted for RCP4.5 (left) and RCP8.5 (right). Units: $GtCO_2$ per yr. Mean of 10,000 Monte-Carlo runs of PAGE-ICE. Source: Supplementary Data 1.

Supplementary Figure 4

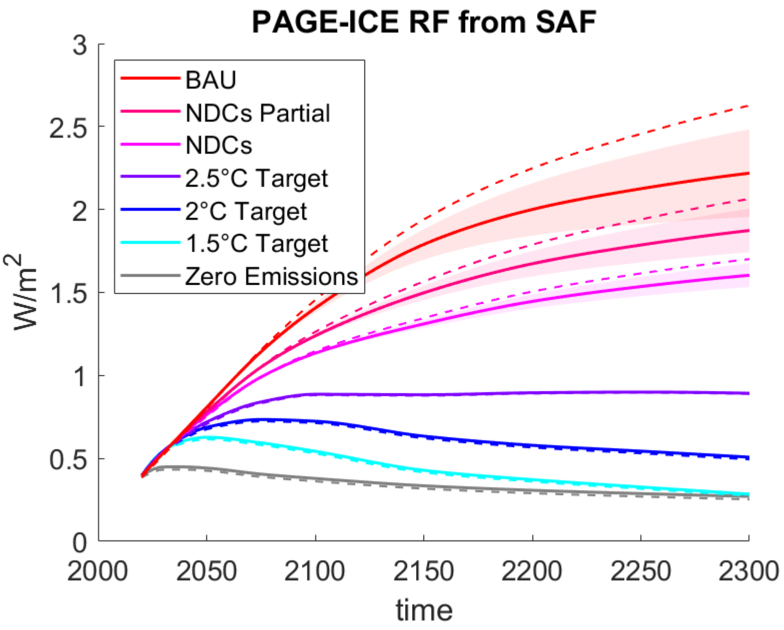

Global equivalent RF from the nonlinear SAF (solid lines: mean; shaded areas: ±1 SD) and cumulative RF corresponding to temperature-invariant SAF of 0.349±0.045 W/m2/°C, which represents average SAF for the period between pre-industrial conditions and the 2xCO2 ECS warming level (dashed lines), plotted for the climate scenarios considered. 100,000 Monte-Carlo runs of PAGE-ICE. Source: Supplementary Data 1.

Supplementary Figure 5

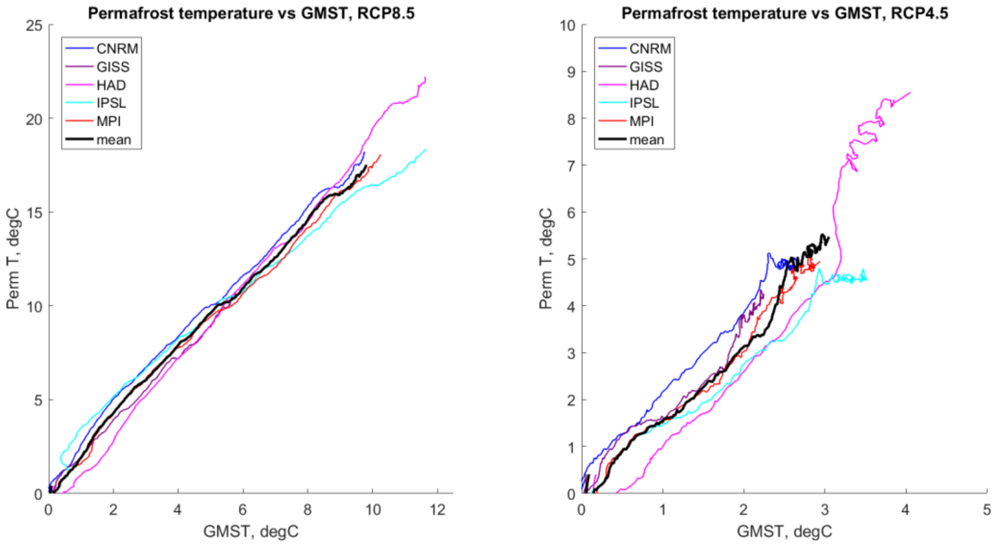

Mean annual surface air temperatures averaged across the permafrost regions (defined according to pre-industrial conditions) as functions of the relevant GMST increases, simulated by the five CMIP5 models used in SiBCASA runs under RCP8.5 and RCP4.5 out to 2300. The slopes of the curves define the permafrost AF. Note the difference in the Y-axis scale between the plots. Source: Supplementary Data 2.

Supplementary Figure 6

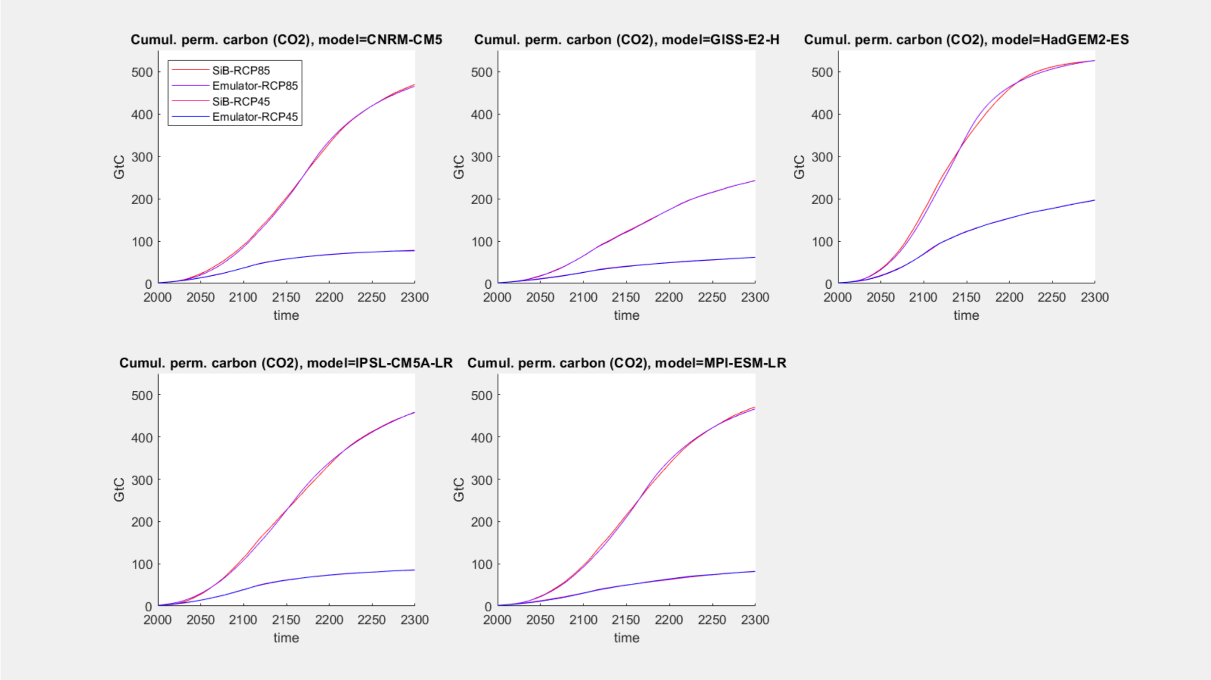

Projections for cumulative land permafrost carbon emissions (CO_2 component) until 2300 under the RCP8.5 and RCP4.5 scenarios, generated by SiBCASA and by our PCF model emulator individually for each of the five CMIP5 models employed in the SiBCASA simulations. In the calibration procedure illustrated by these plots, the emulator was forced by the mean annual GMST projections from the same CMIP5 experiments that were used to conduct the SiBCASA runs. The emulator parameters are from the final iteration. Source: Supplementary Code 2.

Supplementary Figure 7

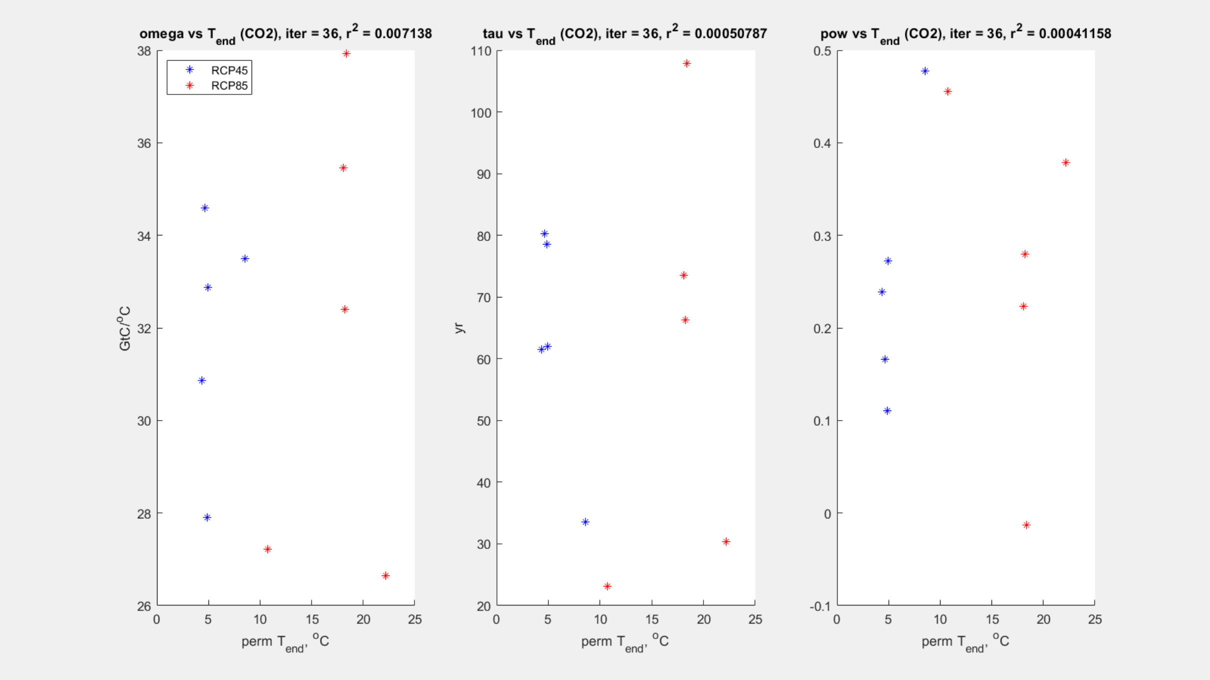

Values of the three emulator parameters $(\omega, \tau, p)_{m,s}$ from the final iteration plotted as functions of the permafrost temperature T_{end} in year 2300 in each SiBCASA run (CO_2 component). The residual inter-scenario biases for the parameters of the dynamic model emulator are described by the correlations that all fall below the required threshold of 0.01. Source: Supplementary Code 2.

Supplementary Figure 8

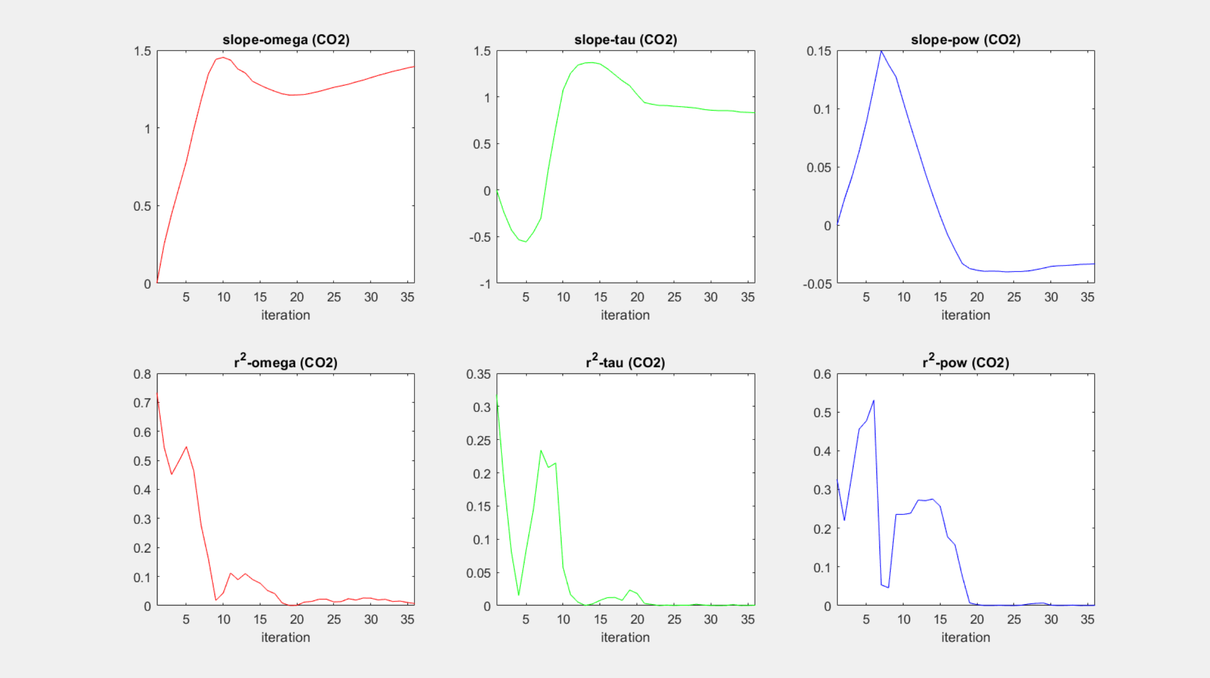

Slopes δ_ω, δ_τ, δ_p defining the corrections to the emulator parameters ω, τ, p in Supplementary Equation 13 (upper row), and the correlation coefficients $R^2\{\omega_{m,s}, T_{m,s}(t_N)\}, R^2\{\tau_{m,s}, T_{m,s}(t_N)\}, R^2\{p_{m,s}, T_{m,s}(t_N)\}$ (lower row), plotted for all the iterations. SiBCASA, CO_2 component. Source: Supplementary Code 2.

Supplementary Figure 9

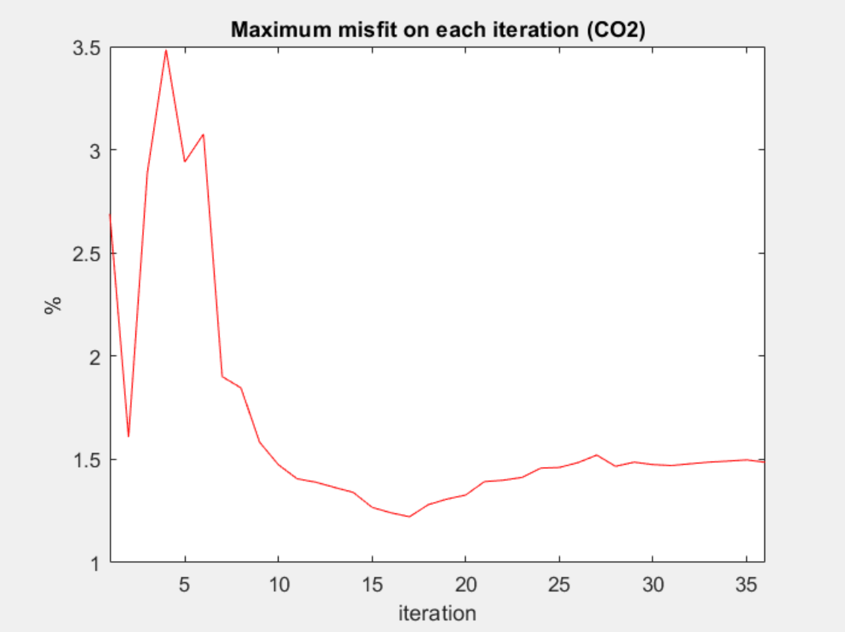

Evolution of the maximum normalised misfit across all the models and scenarios with the iterations. The misfit tends to decrease with the iterations before converging to around 1.5%. SiBCASA, CO_2 component. Source: Supplementary Code 2.

Supplementary Figure 10

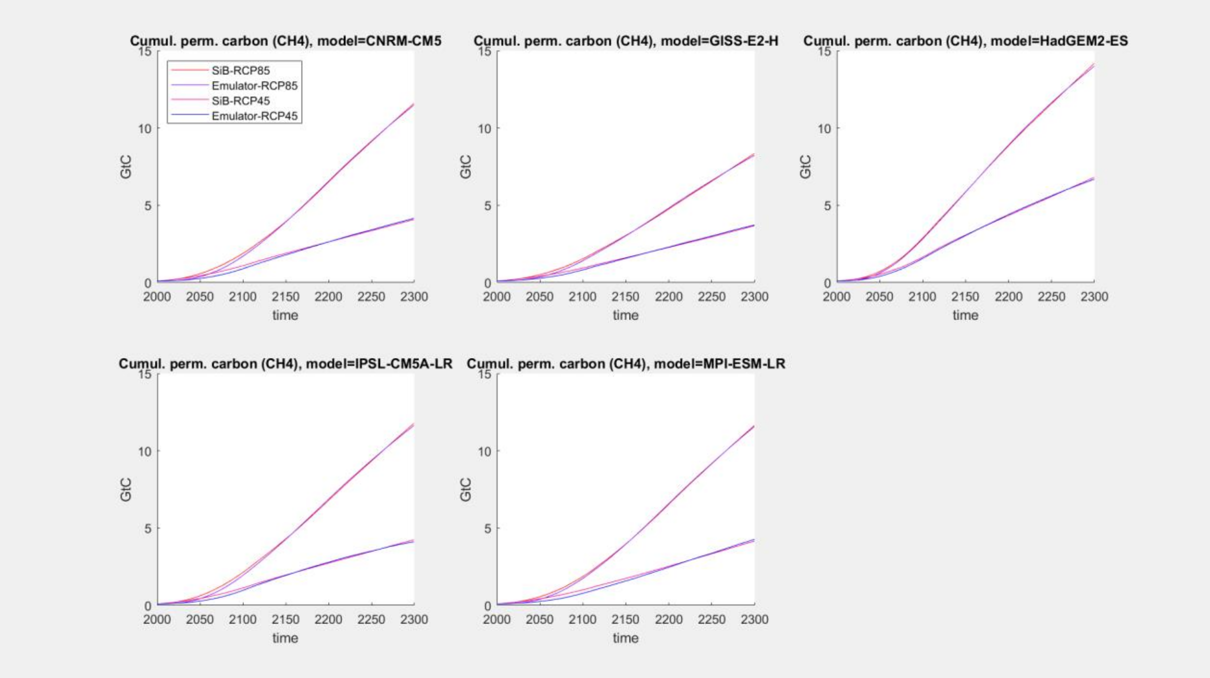

Projections for cumulative land permafrost carbon emissions (methane component) until 2300 under the RCP8.5 and RCP4.5 scenarios, generated by SiBCASA and by our PCF model emulator individually for each of the five CMIP5 models employed in the SiBCASA simulations. In the calibration procedure illustrated by these plots, the emulator was forced by the mean annual GMST projections from the same CMIP5 experiments that were used to conduct the SiBCASA runs. The emulator parameters are from the final iteration. Source: Supplementary Code 3.

Supplementary Figure 11

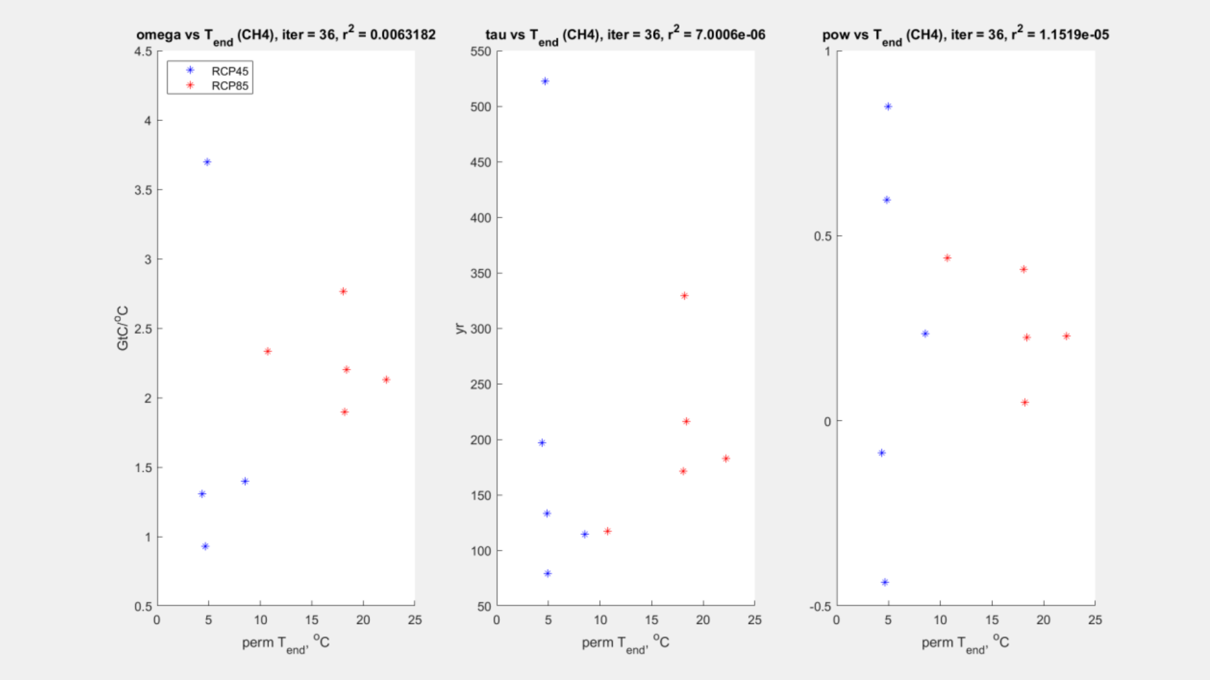

Values of the three emulator parameters $(\omega,\tau,p)_{m,s}$ from the final iteration plotted as functions of the permafrost temperature T_{end} in year 2300 in each SiBCASA run (methane component). The residual inter-scenario biases for the parameters of the dynamic model emulator are described by the correlations that all fall below the required threshold of 0.01. Source: Supplementary Code 3.

Supplementary Figure 12

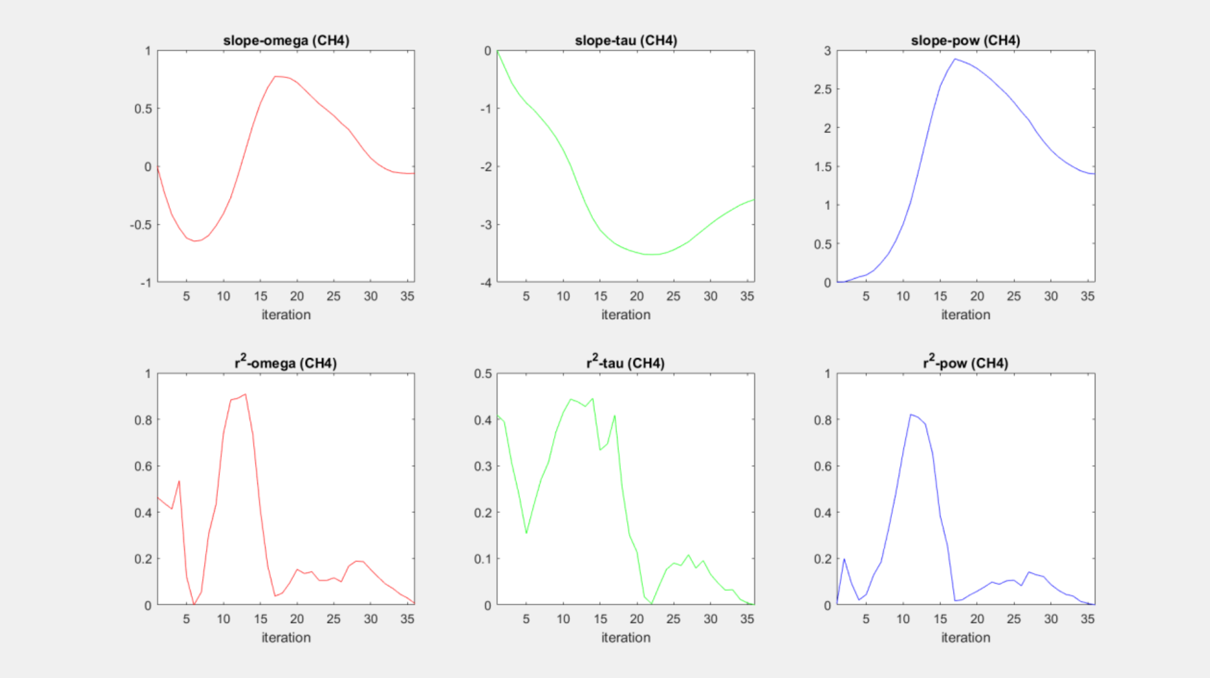

Slopes δ_ω, δ_τ, δ_p defining the corrections to the emulator parameters ω,τ,p in Supplementary Equation 13 (upper row), and the correlation coefficients $R^2\{\omega_{m,s},T_{m,s}(t_N)\},R^2\{\tau_{m,s},T_{m,s}(t_N)\},R^2\{p_{m,s},T_{m,s}(t_N)\}$ (lower row), plotted for all the iterations. SiBCASA, methane component. Source: Supplementary Code 3.

Supplementary Figure 13

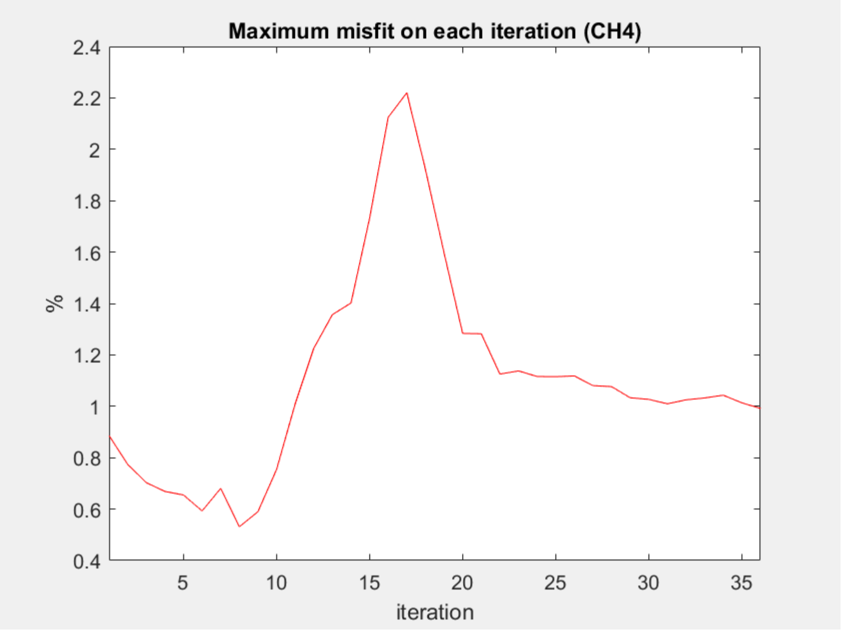

Evolution of the maximum normalised misfit across all the models and scenarios with the iterations. The misfit converges to around 1%. SiBCASA, methane component. Source: Supplementary Code 3.

Supplementary Figure 14

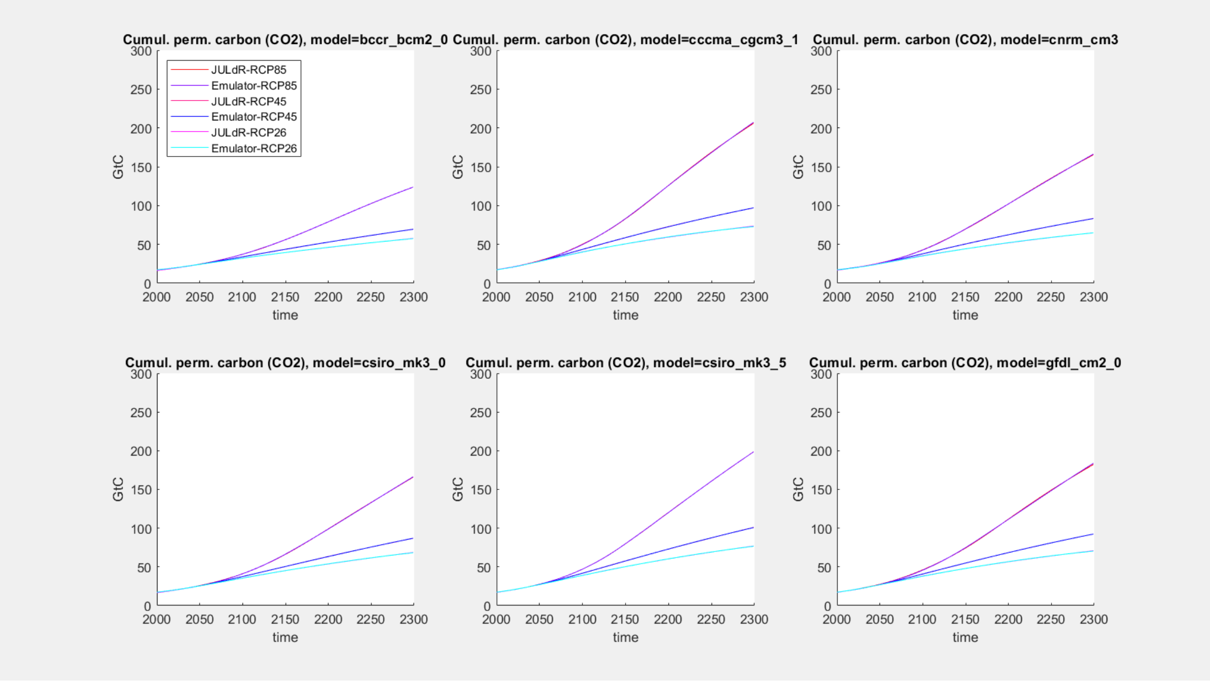

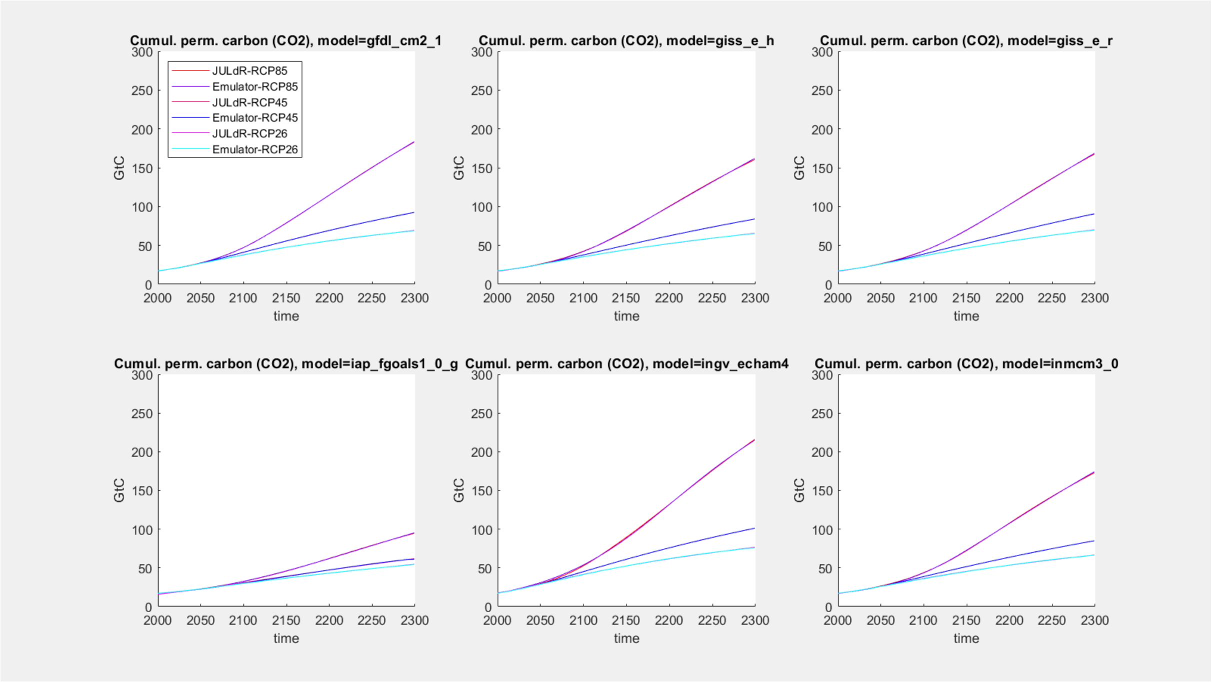
Cumul. perm. carbon (CO2), model=gfdl_cm2_1
Cumul. perm. carbon (CO2), model=giss_e_h
Cumul. perm. carbon (CO2), model=giss_e_r
Cumul. perm. carbon (CO2), model=iap_fgoals1_0_g
Cumul. perm. carbon (CO2), model=ingv_echam4
Cumul. perm. carbon (CO2), model=inmcm3_0
JULdR-RCP85
Emulator-RCP85
JULdR-RCP45
Emulator-RCP45
JULdR-RCP26
Emulator-RCP26
GtC
time

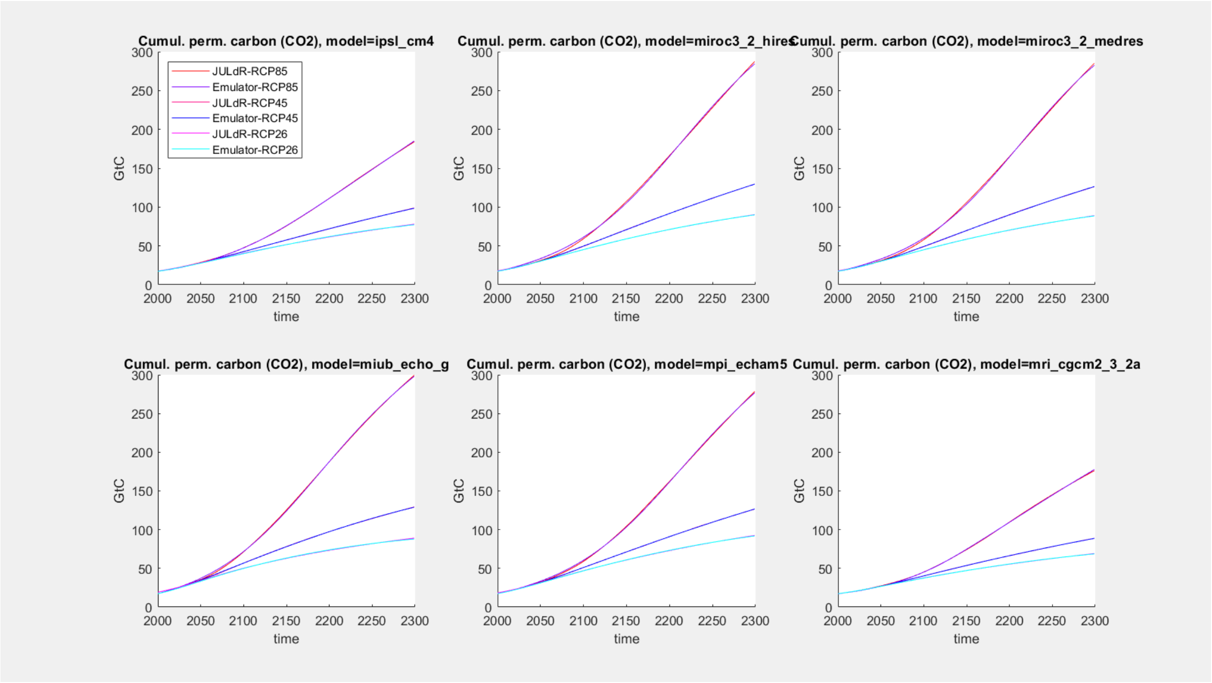
Cumul. perm. carbon (CO2), model=ipsl_cm4
Cumul. perm. carbon (CO2), model=miroc3_2_hires
Cumul. perm. carbon (CO2), model=miroc3_2_medres
Cumul. perm. carbon (CO2), model=miub_echo_g
Cumul. perm. carbon (CO2), model=mpi_echam5
Cumul. perm. carbon (CO2), model=mri_cgcm2_3_2a
JULdR-RCP85
Emulator-RCP85
JULdR-RCP45
Emulator-RCP45
JULdR-RCP26
Emulator-RCP26
GtC
time

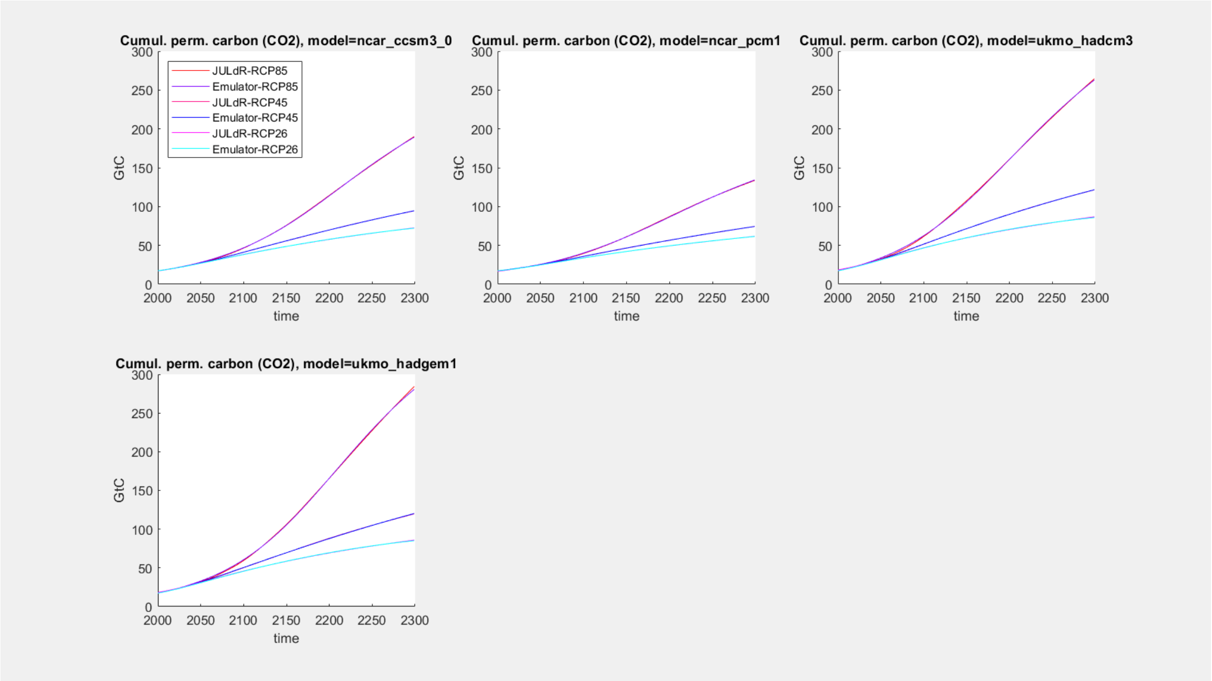

Projections for cumulative land permafrost carbon emissions (CO_2 component) until 2300 under the RCP8.5, RCP4.5 and RCP2.6 scenarios, generated by JULES and by our PCF model emulator individually for each of the 22 CMIP3 models employed in the JULES simulations. In the calibration procedure illustrated by these plots, the emulator was forced by the mean annual GMST projections from the same CMIP3 experiments that were used to conduct the JULES runs. The emulator parameters are from the final iteration. Source: Supplementary Code 4.

Supplementary Figure 15

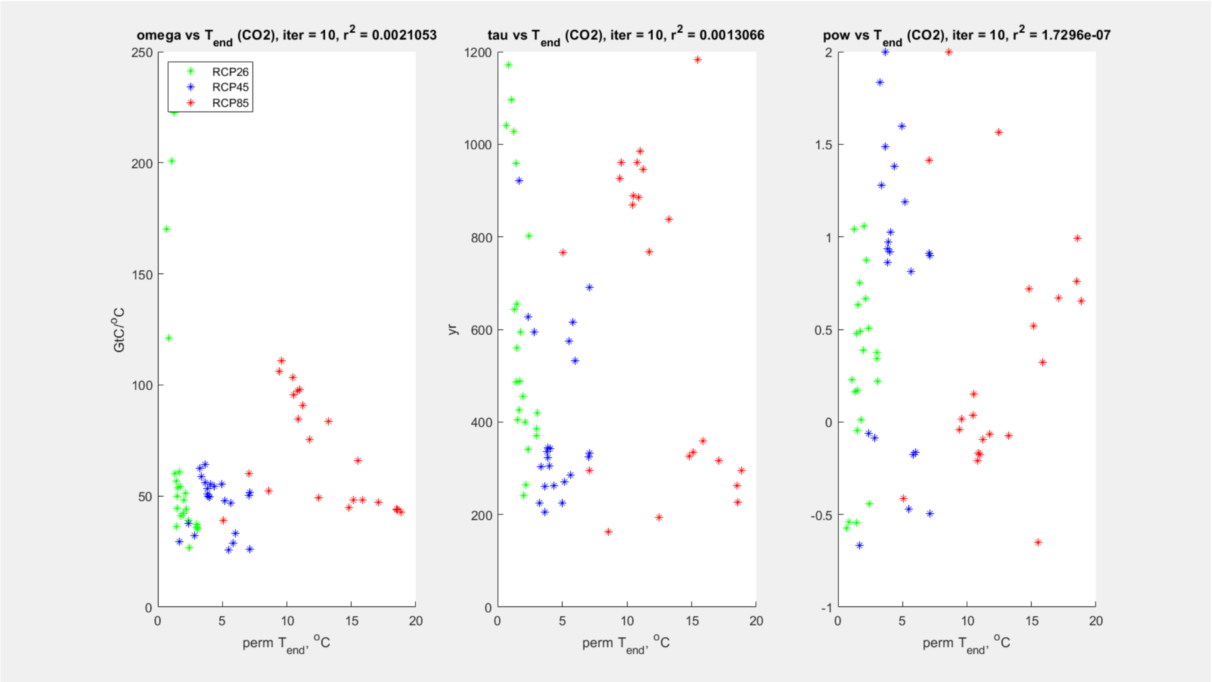

Values of the three emulator parameters $(\omega, \tau, p)_{m,s}$ from the final iteration plotted as functions of the permafrost temperature T_{end} in year 2300 in each JULES run (CO_2 component). The residual inter-scenario biases for the parameters of the dynamic model emulator are described by the correlations that all fall below the required threshold of 0.01. Source: Supplementary Code 4.

Supplementary Figure 16

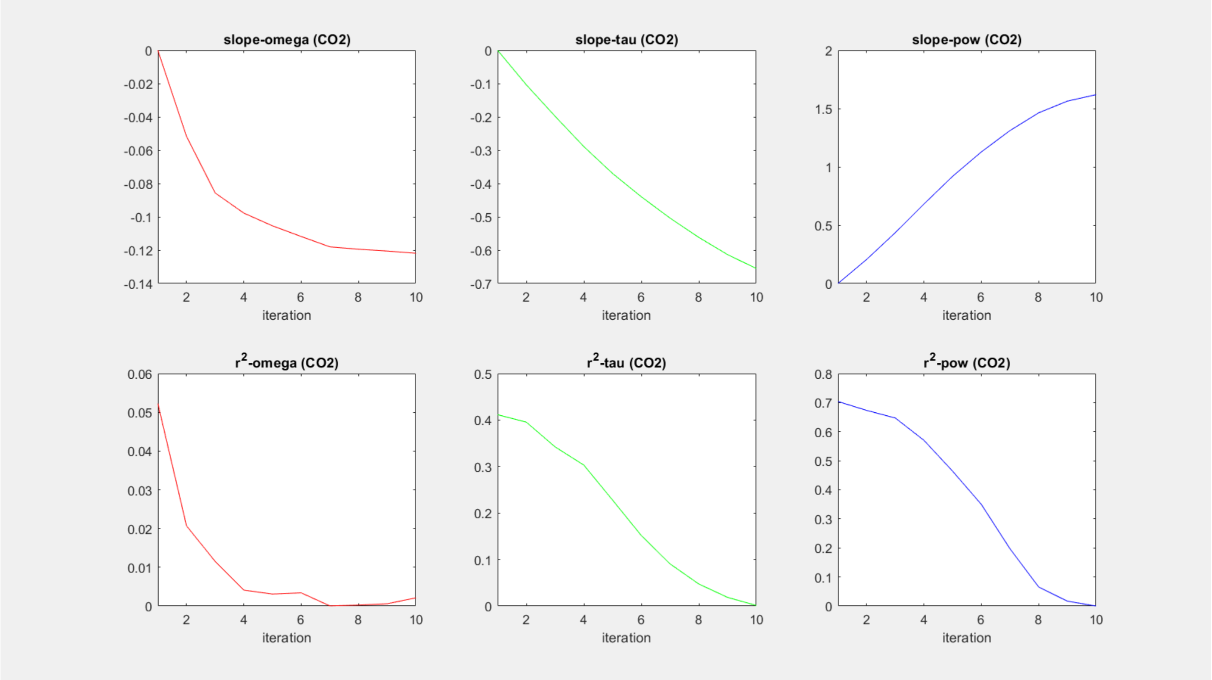

Slopes δ_ω, δ_τ, δ_p defining the corrections to the emulator parameters ω, τ, p in Supplementary Equation 13 (upper row), and the correlation coefficients $R^2\{\omega_{m,s}, T_{m,s}(t_N)\}, R^2\{\tau_{m,s}, T_{m,s}(t_N)\}, R^2\{p_{m,s}, T_{m,s}(t_N)\}$ (lower row), plotted for all the iterations. JULES, CO_2 component. Source: Supplementary Code 4.

Supplementary Figure 17

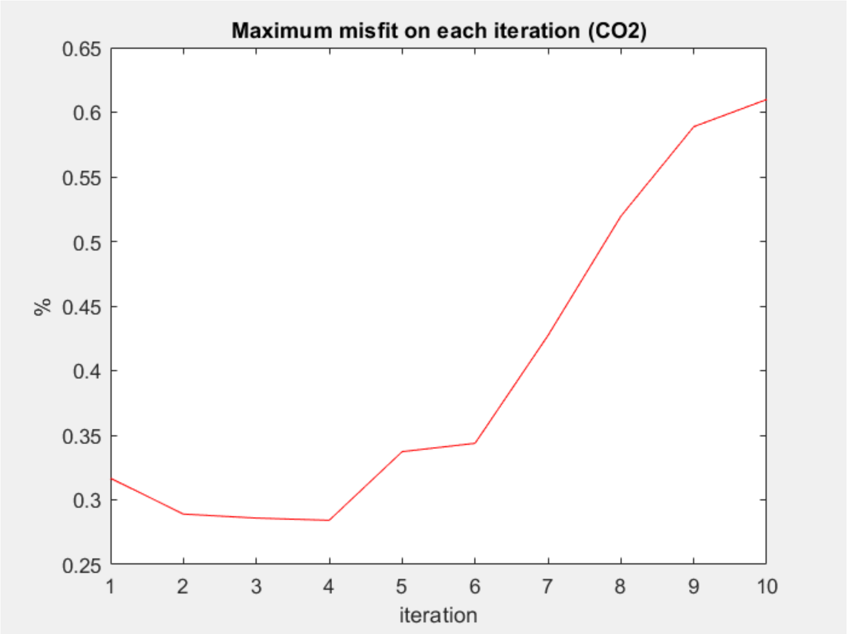

Evolution of the maximum normalised misfit across all the models and scenarios with the iterations. While the misfit increases with the iterations, the increase slows down and the misfit remains at around 0.6% when the required residual inter-scenario bias targets are met for all the three emulator parameters. JULES, CO2 component. Source: Supplementary Code 4.

Supplementary Figure 18

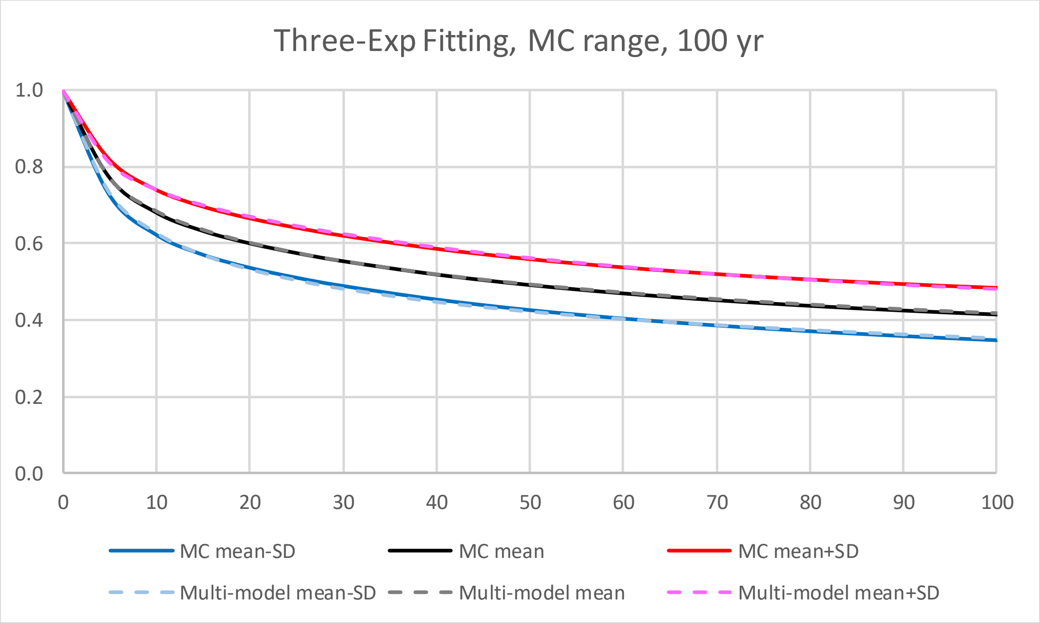

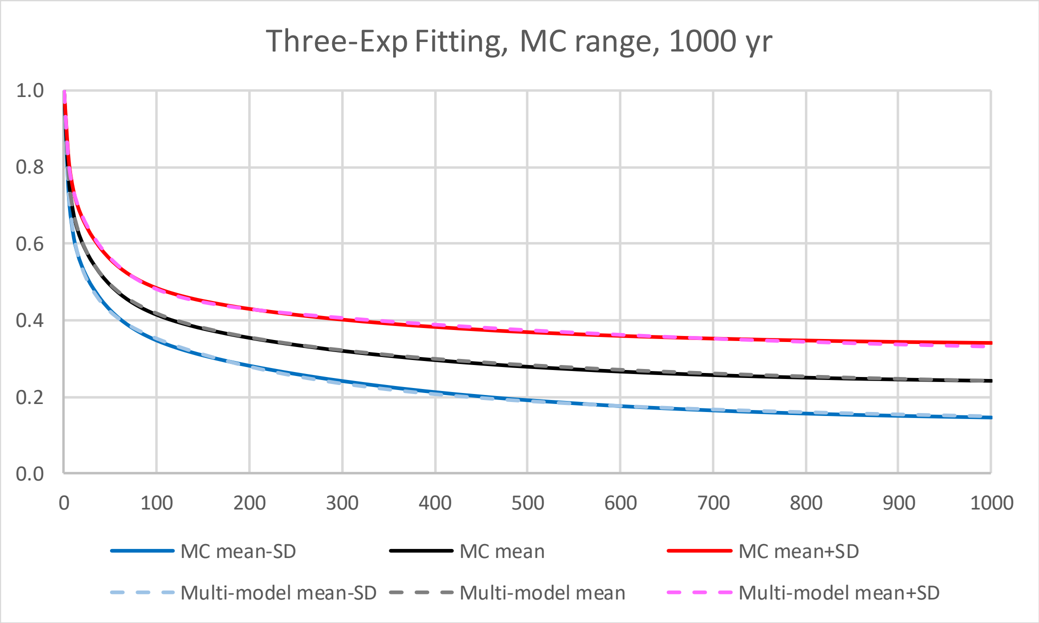

Multi-model mean and ± 1SD range of the CO_2 response function from Joos et al. (2013) (dashed lines), and the corresponding simulated Monte-Carlo (MC) mean and ± 1SD range of the fitted response function $f(\tau)$ with uncertain parameters $\{a_n, \tau_n\}$ from Supplementary Equation 1 (solid lines), plotted on the timescale of 100 (top) and 1000 (bottom) years. Source: Supplementary Data 11.

Supplementary Figure 19

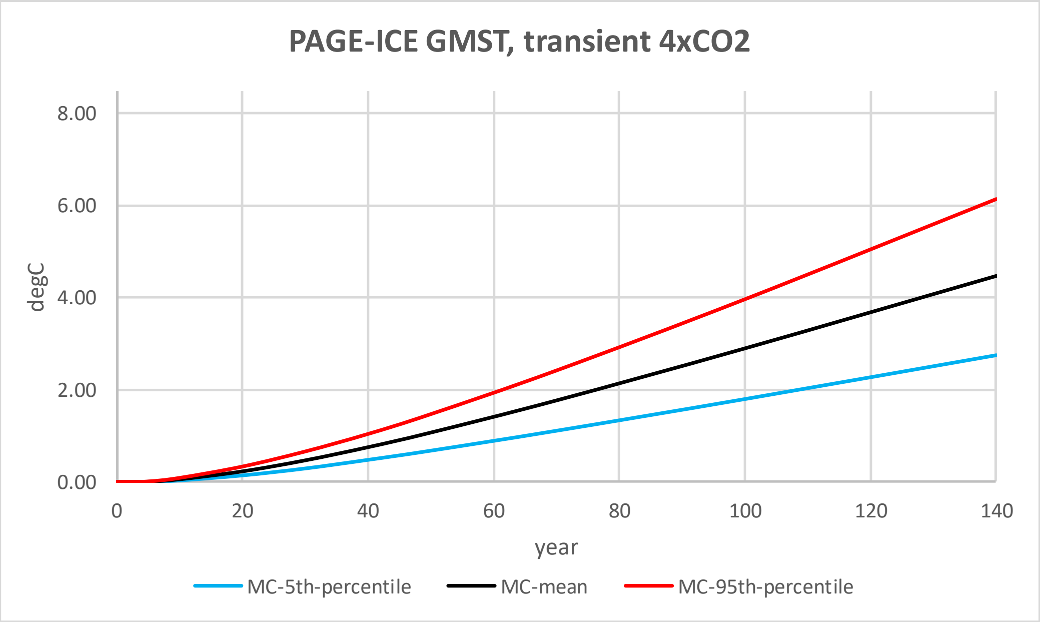

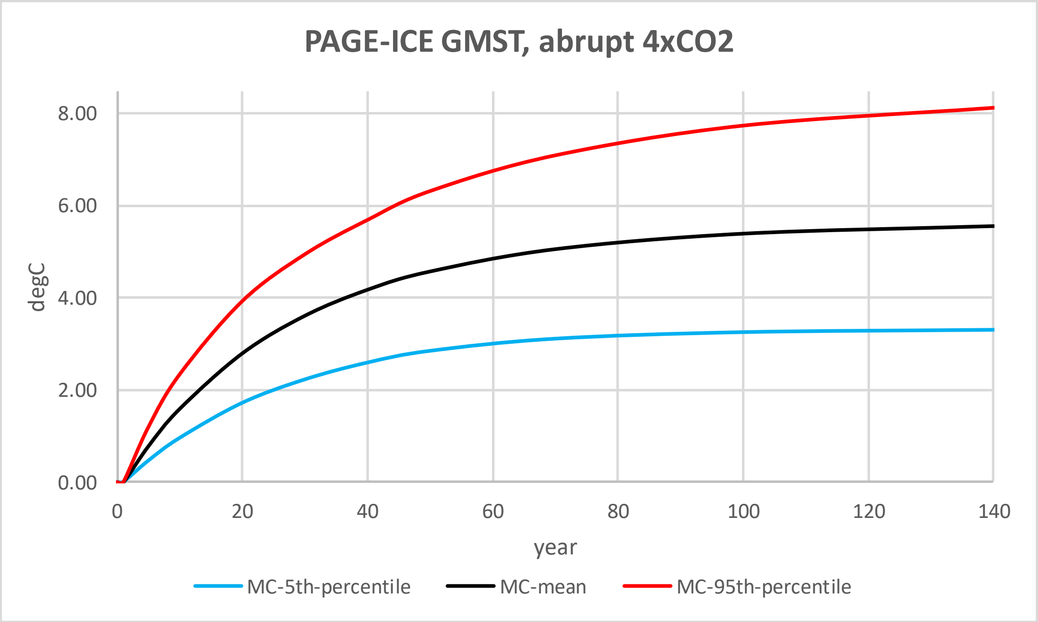

GMST projections (5-95% range and mean) for the transient 2xCO2 and 4xCO2 (top) and abrupt 4xCO2 (bottom) experiments in PAGE-ICE. Source: Supplementary Code 1.

Supplementary Figure 20

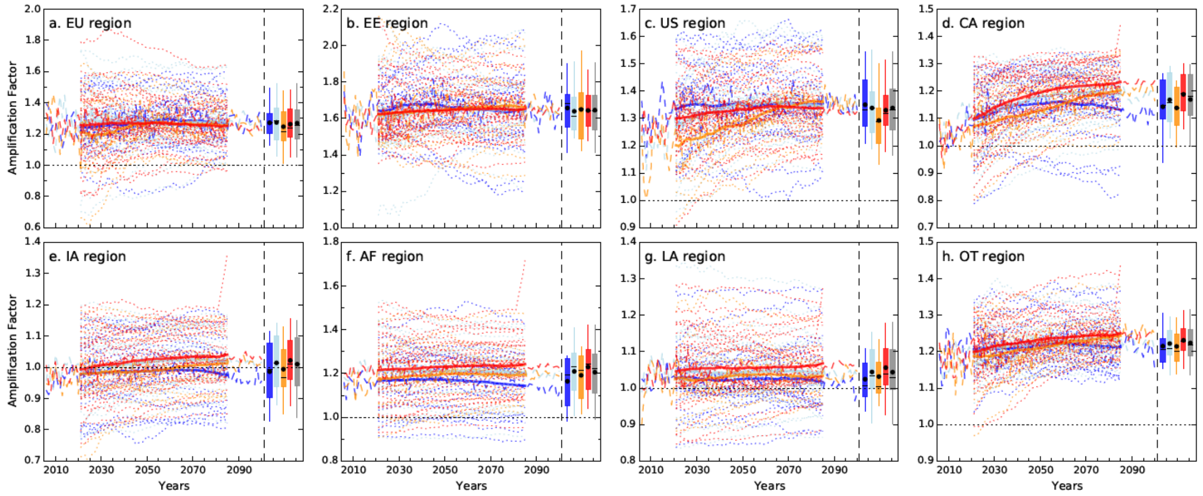

Amplification factor 30-year running mean time series from 2021 to 2085 for the 8 regions of the PAGE model: (a) European Union, (b) Russia and the rest Eastern Europe (non-EU), (c) United States, (d) China and South-East Asia, (e) India and South Asia, (f) Africa, (g) Latin America and (h) Other OECD countries. The multi-model mean is represented by a solid line and individual models by dotted lines for the RCP2.6 (blue), RCP4.5 (cyan) RCP6.0 (orange) and RCP8.5 (red), respectively. The combined uncertainty over the CMIP5 ensemble and over the time range is represented by the box, whiskers and line, which indicate the interquartile range, 95% range and median, respectively. The corresponding means are indicated by dots. The multi-scenario mean (RCP-2.6-4.5-8.5) is shown in grey. Source: Supplementary Code 13.

Supplementary Figure 21

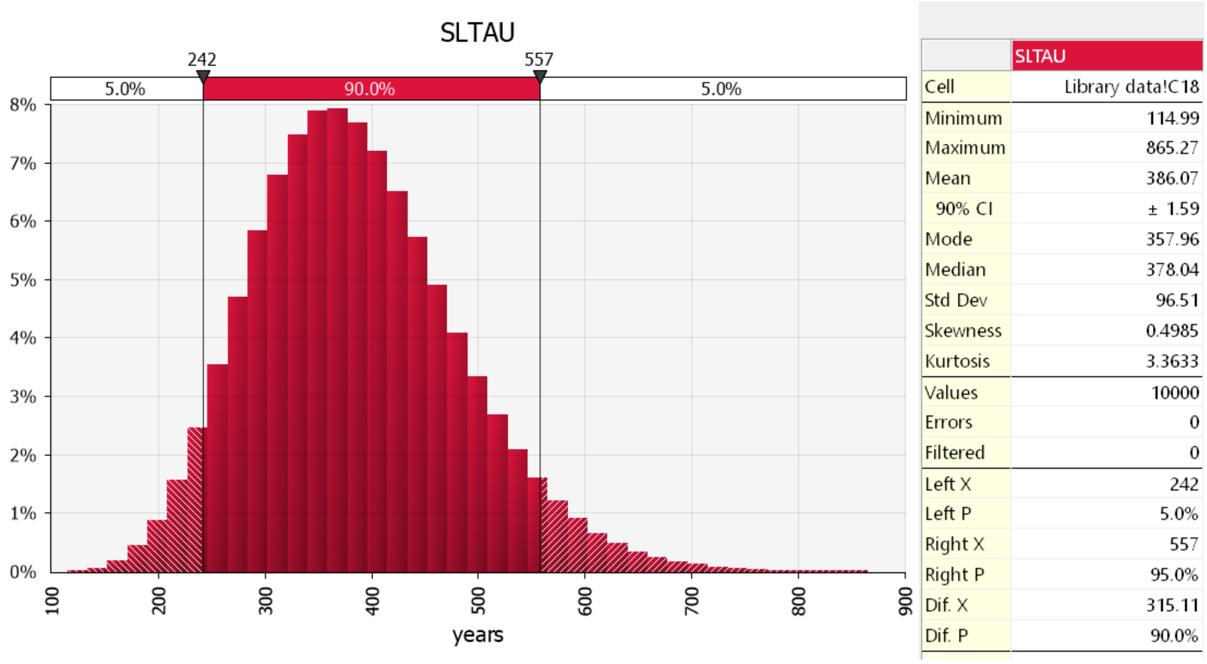

Gamma distribution for the SLR lag parameter τ_{SLR} calibrated using the Nauels et al. (2017) SLR projections. Source: Supplementary Code 1.

Supplementary Figure 22

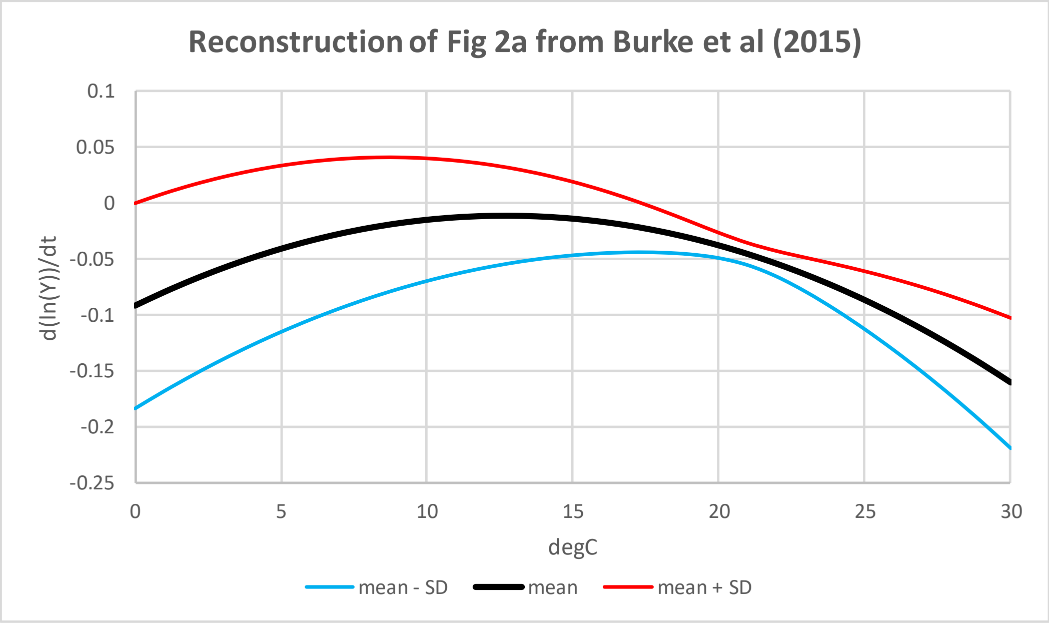

Economic impact function from Burke et al. expressed in terms of the absolute temperature in °C, reconstructed using 10,000 Monte-Carlo simulations of Supplementary Equation 8. Source: Supplementary Data 12.

Supplementary Figure 23

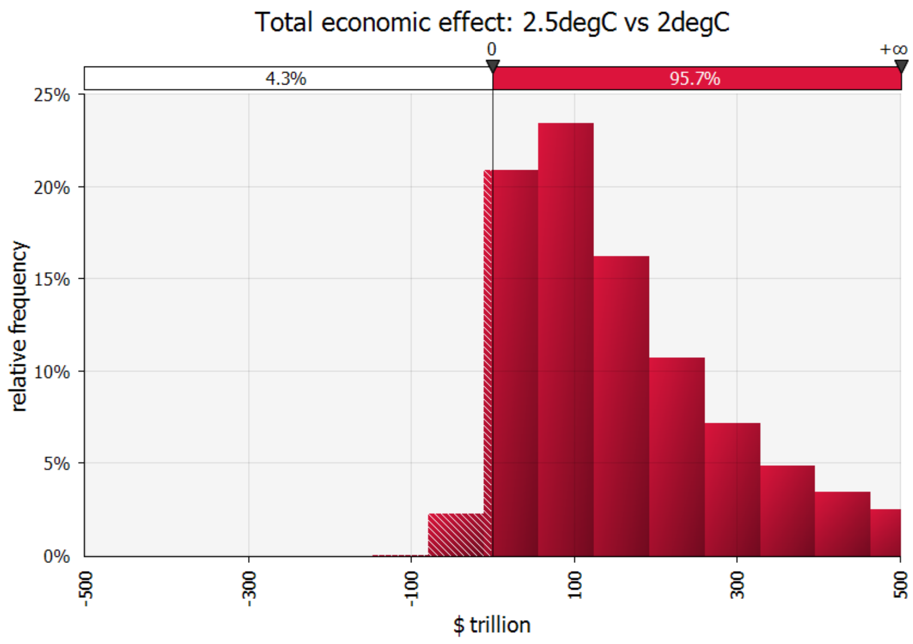

	Total economic effect / Total
Cell	Totals!L39
Minimum	-147.10
Maximum	6,646.39
Mean	217.86
90% CI	± 1.42
Mode	41.97
Median	136.97
Std Dev	272.18
Skewness	3.9783
Kurtosis	33.3408
Values	100000
Errors	0
Filtered	0
Left X	0
Left P	4.3%
Right X	+∞
Right P	100.0%
Dif. X	+∞
Dif. P	95.7%

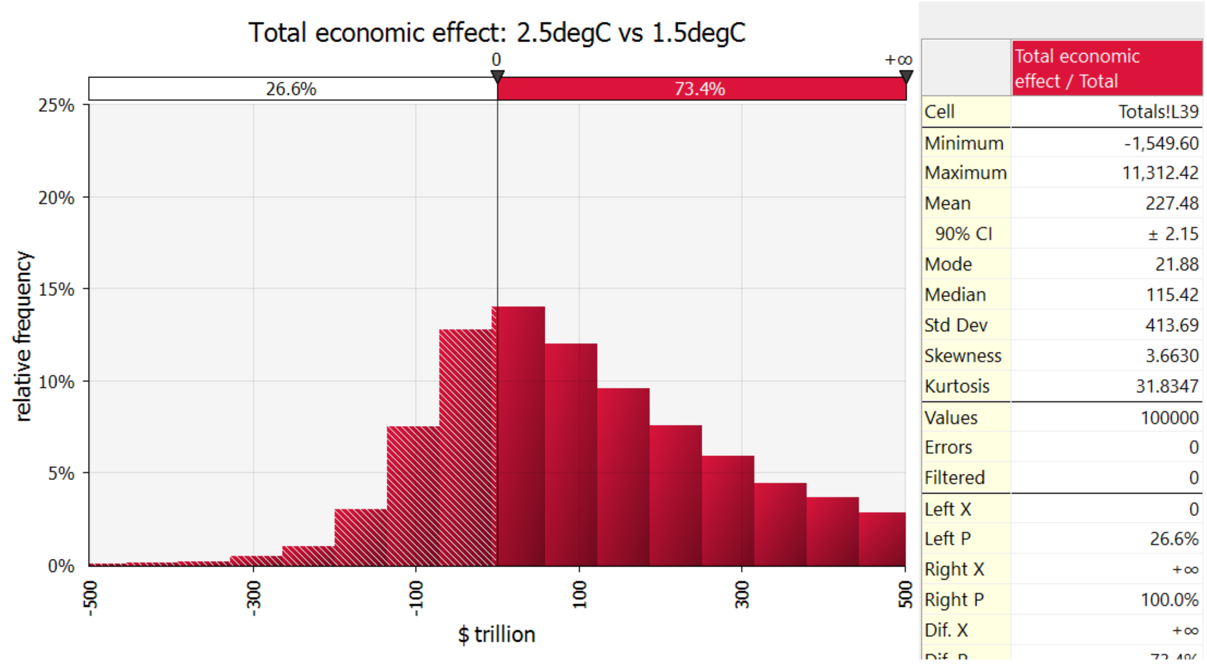

Probability density functions of the differences between the total economic effects of climate change with the nonlinear PCF and SAF for the following pairs of scenarios: 2.5°C and 2°C targets (top), and 2.5°C and 1.5°C targets (bottom). Horizontal axes units: $trillion = 1 million millions, NPV until 2300, equity-weighted, PTP-discounted. 100,000 Monte-Carlo runs of PAGE-ICE. Source: Supplementary Data 1.

Supplementary Table 1

*CMIP5 models used for computing the SAF based on the ALL/CLR method by Winton. The models marked with **(P)** were also used to drive SiBCASA LSM simulations to calculate the PCF.*

Modeling Center (or Group)	Institute ID	Model Name
Commonwealth Scientific and Industrial Research Organization (CSIRO) and Bureau of Meteorology (BOM), Australia	CSIRO-BOM	ACCESS1.3
Beijing Climate Center, China Meteorological Administration	BCC	BCC-CSM1.1
Canadian Centre for Climate Modelling and Analysis	CCCMA	CanESM2
National Center for Atmospheric Research	NCAR	CCSM4
Community Earth System Model Contributors	NSF-DOE-NCAR	CESM1(CAM5)
Centre National de Recherches Météorologiques / Centre Européen de Recherche et Formation Avancée en Calcul Scientifique	CNRM-CERFACS	CNRM-CM5 **(P)**
Commonwealth Scientific and Industrial Research Organization in collaboration with	CSIRO-QCCCE	CSIRO-Mk3.6.0

Queensland Climate Change Centre of Excellence		
NOAA Geophysical Fluid Dnamics Laboratory	NOAA GFDL	GFDL-CM3
NASA Goddard Institute for Space Studies	NASA GISS	GISS-E2-H **(P)**
Met Office Hadley Centre (additional HadGEM2-ES realizations contributed by Instituto Nacional de Pesquisas Espaciais)	MOHC (additional realizations by INPE)	HadGEM2-ES **(P)**
Institute for Numerical Mathematics	INM	INM-CM4
Institut Pierre-Simon Laplace	IPSL	IPSL-CM5A-LR **(P)**
Japan Agency for Marine-Earth Science and Technology, Atmosphere and Ocean Research Institute (The University of Tokyo), and National Institute for Environmental Studies	MIROC	MIROC-ESM
Max-Planck-Institut für Meteorologie (Max Planck Institute for Meteorology)	MPI-M	MPI-ESM-LR **(P)**
Meteorological Research Institute	MRI	MRI-CGCM3
Norwegian Climate Centre	NCC	NorESM1-ME

Supplementary Table 2

Results from the final iteration for the slopes δ_ω, δ_τ, δ_p defining the temperature-dependent corrections to ω, τ, p, together with the residual correlations. SiBCASA, CO_2 component. The corresponding probability distributions defined by the sets of values $(\omega, \tau, p)_{m,s}$ from the final iteration are given in Supplementary Table 5.

Parameter	Value	Units
highest perm temperature, T_{max}	22.2	°C
final iteration number	36	
max_misfit, $\max(\varepsilon_{m,s})\ \forall\, m, s$	0.01486	fraction of 1
slope_omega, δ_ω	1.39535	dimensionless
r2_omega, $R^2\{\omega_{m,s}, T_{m,s}(t_N)\}$	0.00714	fraction of 1
slope_tau, δ_τ	0.82921	dimensionless
r2_tau, $R^2\{\tau_{m,s}, T_{m,s}(t_N)\}$	0.00051	fraction of 1
slope_pow, δ_p	-0.03335	dimensionless
r2_pow, $R^2\{p_{m,s}, T_{m,s}(t_N)\}$	0.00041	fraction of 1

Supplementary Table 3

Results from the final iteration for the slopes δ_ω, δ_τ, δ_p defining the temperature-dependent corrections to ω, τ, p, together with the residual correlations. SiBCASA, methane component. The corresponding probability distributions defined by the sets of values $(\omega, \tau, p)_{m,s}$ from the final iteration are given in Supplementary Table 5.

Parameter	Value	Units
highest perm temperature, T_{max}	22.2	°C
final iteration number	36	
max_misfit, $\max(\varepsilon_{m,s})\ \forall\, m, s$	0.00992	fraction of 1
slope_omega, δ_ω	-0.06163	dimensionless
r2_omega, $R^2\{\omega_{m,s}, T_{m,s}(t_N)\}$	0.00632	fraction of 1
slope_tau, δ_τ	-2.57522	dimensionless
r2_tau, $R^2\{\tau_{m,s}, T_{m,s}(t_N)\}$	0.00001	fraction of 1
slope_pow, δ_p	1.39921	dimensionless
r2_pow, $R^2\{p_{m,s}, T_{m,s}(t_N)\}$	0.00001	fraction of 1

Supplementary Table 4

Results from the final iteration for the slopes δ_ω, δ_τ, δ_p defining the temperature-dependent corrections to ω, τ, p, together with the residual correlations. JULES, CO_2 component. The corresponding probability distributions defined by the sets of values $(\omega, \tau, p)_{m,s}$ from the final iteration are given in Supplementary Table 5.

Parameter	Value	Units
highest perm temperature, T_{max}	18.7	°C
final iteration number	10	
max_misfit, $\max(\varepsilon_{m,s})\ \forall\, m, s$	0.00610	fraction of 1
slope_omega, δ_ω	-0.12187	dimensionless
r2_omega, $R^2\{\omega_{m,s}, T_{m,s}(t_N)\}$	0.00211	fraction of 1
slope_tau, δ_τ	-0.65501	dimensionless
r2_tau, $R^2\{\tau_{m,s}, T_{m,s}(t_N)\}$	0.00131	fraction of 1
slope_pow, δ_p	1.61888	dimensionless
r2_pow, $R^2\{p_{m,s}, T_{m,s}(t_N)\}$	0.00000	fraction of 1

Supplementary Table 5

Uncertainty ranges for the statistical parameters ω, τ, p defining the PCF emulator in PAGE-ICE, together with the permafrost AF, which are based on the fitting to the SiBCASA simulations with multiple GCMs under multiple climate scenarios. The results are given separately for the CO_2 and methane components.

PCF emulator parameters	Min	Mod	Max	Units
$\mathbf{AF_p}$ (perm. ampl. factor)	1.43	1.84	2.33	
$\boldsymbol{\omega}$ (equilib. sensitivity), CO_2	28,191	31,940	35,688	MtonC/degC
$\boldsymbol{\tau}$ (time lag), CO_2	35.49	61.69	87.89	Yr
$\boldsymbol{p}$ (power), CO_2	0.11	0.26	0.41	
$\boldsymbol{\omega}$ (equilib. sensitivity), methane	1,240	2,294	3,348	MtonC/degC
$\boldsymbol{\tau}$ (time lag), methane	75.19	206.29	337.38	Yr
$\boldsymbol{p}$ (power), methane	-0.11	0.25	0.61	

Supplementary Table 6

Uncertainty ranges for the statistical parameters ω, τ, p defining the PCF emulator in PAGE-ICE, together with the permafrost AF, which are based on the fitting to the JULES simulations with multiple GCMs under multiple climate scenarios.

PCF emulator parameters	Min	Mod	Max	Units
AF_p (perm. ampl. factor)	1.71	1.94	2.16	
ω (equilib. sensitivity), CO_2	24,727	61,868	99,009	MtonC/degC
τ (time lag), CO_2	253	544	835	Yr
p (power), CO_2	-0.23	0.46	1.14	

Supplementary Table 7

Calibration parameters of the CO_2 cycle in PAGE-ICE obtained from statistical fitting to the multi-model CO_2 response function results.

CO_2 cycle parameters	Min	Mod	Max	Units
Percentage of CO2 long-term ocean uptake (a_1)	4.3	23.0	41.6	%
Percentage of CO2 short-term ocean uptake (a_2)	23.1	26.6	30.1	%
Percentage of CO2 land uptake (a_3)	11.4	27.0	42.5	%
Timescale of CO2 long-term ocean uptake (τ_1)	248.9	312.5	376.2	years
Timescale of CO2 short-term ocean uptake (τ_2)	25.9	34.9	43.9	years
Timescale of CO2 land uptake (τ_3)	2.8	4.3	5.7	years

Supplementary Table 8

Calibration of the climate sensitivity parameters (TCR, ECS) and the e-folding feedback response time of the upper ocean layers (FRT) to increased RF in PAGE-ICE.

Climate sensitivity parameters	Min	Mod	Max	Units
TCR	0.8	1.8	2.7	°C
FRT	10	20	55	yr
ECS (function of TCR and FRT)	1.02	2.56	5.95	°C

Supplementary Table 9

GMST projections (5-95% range and mean) for the transient 2xCO2 and 4xCO2 and abrupt 4xCO2 experiments in PAGE-ICE after 70 and 140 years.

Transient 4xCO2	70 years	140 years
5th percentile	1.1	2.8
Mean	1.8	4.5
95th percentile	2.4	6.2

Abrupt 4xCO2	70 years	140 years
5th percentile	3.1	3.3
Mean	5.1	5.6
95th percentile	7.1	8.1

Supplementary Table 10

Probability ranges for the AFs in the 8 regions of PAGE-ICE based on the CMIP5 multi-model and multi-RCP data over the 21st century (moving 30-year climatological windows). Units: °C regional per °C global.

PAGE-ICE Regions	Min	Mod	Max
EU	1.05	1.23	1.53
US	1.16	1.32	1.54
OT	1.14	1.21	1.31
EE	1.41	1.64	1.9
CA	1	1.21	1.3
IA	0.84	1.04	1.15
AF	0.99	1.22	1.42
LA	0.9	1.04	1.18

Supplementary Table 11

Calibration of the SLR driver in PAGE-ICE. The probability parameters for the time constant τ_{SLR} of SLR are obtained from 100,000 Monte-Carlo simulations.

SLR driver parameters	Min	Mod	Max	Units
Sensitivity to GMST changes	0.7	1.5	3	m/°C
Asymptote for pre-industrial	0.5	1	1.5	m
Time constant (Gamma distrib.)	115	362	865	yr

Supplementary Table 12

Uncertainty in BAU parameter for the four main policy gases in PAGE-ICE. Units: emission levels measured as percentage changes from the corresponding RCP8.5 emissions levels in 2100.

Policy gases	Min	Mod	Max
CO_2	−50%	−22%	6%
CH_4	−67%	−30%	6%
N_2O	−20%	−7%	6%
Linear[i]	−50%	0%	+50%

Supplementary Table 13

[i] This category of GHG has multiple components with no clear direction of change from RCP8.5 to RCP6.0 in 2100, so substantial changes in both directions are possible.

Calibration of the parameters defining the discontinuity impact sector in PAGE-ICE.

Discontinuity parameters	Min	Mod	Max	Units
**Tolerable GMST level**	1	1.5	2	°C
**Losses if occurred**	1	3	5	% of GDP
**Timescale of the losses**	10	20	30	Yr

Supplementary Table 14

PAGE-ICE calibration of MAC curves and experience-based learning rates for CO_2 (first five parameters), and additional adjustments to the MAC curves for all the GHG expected to occur by 2100 relative to 2015 (last four parameters).

CO_2 MAC curve parameters	Min	Mod	Max	Units
Cutbacks at negative cost	0	10	20	% of emiss.
Most negative cost cutback	−150	−100	−50	$ / Mton
Max cutbacks at positive cost	50	60	70	% of emiss.
Maximum cutback cost	100	150	200	$ / Mton
Learning for 2xExperience stock	0.05	0.2	0.35	fraction drop
**General MAC curve evolution**	**Min**	**Mod**	**Max**	**Units**
Cutback at negative cost in 2100	0.6	0.9	1.15	vs 2015
Most negative cost in 2100	0.8	0.9	1.1	vs 2015
Maximum cutback in 2100	1	1.1	1.25	vs 2015
Autonomous change in 2100	0.6	0.65	0.7	vs 2015

Supplementary Table 15

Main specification values of the regression parameters β_1 and β_2 for the economic impact function from Burke et al. (2015) (see their Extended Data Table 1, column 1).

Parameter	mean	SD	units
**Temp. coefficient, β_1**	0.0127	0.0038	1/yr per ºC
**Temp. squared coefficient, β_2**	-0.0005	0.0001	1/yr per (ºC)^2

Supplementary Table 16

PAGE-ICE calibration of climate-driven economic impacts based on the Burke et al. study (short-run specification with one-year regressions), including the mean regional population-weighted temperatures in the 8 PAGE regions corresponding to the 1979-2005 climatology (CMIP5 base period).

Consumption share of GDP	75	80	85	% of GDP
**Linear temperature coefficient, γ_1**	-1.40E-02	-8.30E-03	-2.62E-03	1/yr per ºC
**Quadratic temperature coefficient, γ_2**	-6.00E-04	-5.00E-04	-4.00E-04	1/yr per (ºC)^2
**Impacts saturate beyond**	15	20	25	% consum.
**Mean regional temperatures, 1979-2005 climatology:**				
**EU**	6.762315	10.1222	13.48209	ºC

US	9.542101	13.42862	17.31514	ºC
OT (Other OECD)	9.075961	12.06335	15.05075	ºC
EE (FSU)	3.013205	7.113213	11.21322	ºC
CA	12.23304	15.01296	17.79287	ºC
IA	23.38633	24.94998	26.51362	ºC
AF (Af & ME)	20.18669	21.89225	23.59781	ºC
LA	19.48468	21.1204	22.75611	ºC

Supplementary Table 17

Complete list of the uncertain parameters in PAGE-ICE. Source: Supplementary Code 1 (PAGE-ICE v6.22).

Parameter	mean	Min	mod	max	units
**Discount rates and base year climatology**					
PTP rate	1.033	0.1	1	2	% per year
Elasticity of utility	1.167	0.5	1	2	
Base year cumulative CO2 emissions	2035	1833	2035	2237	Gton CO2
Base year GMST anomaly from pre-ind.	0.95	0.90	0.95	0.99	degC
**Absolute GMST in each region, population-weighted, CMIP5 base period climatology**					
EU	10.12	6.76	10.12	13.48	degC
US	13.43	9.54	13.43	17.32	degC
Other OECD	12.06	9.08	12.06	15.05	degC
FSU	7.11	3.01	7.11	11.21	degC
China & CP Asia	15.01	12.23	15.01	17.79	degC
India and SE Asia	24.95	23.39	24.95	26.51	degC
Africa and ME	21.89	20.19	21.89	23.60	degC
Latin America	21.12	19.48	21.12	22.76	degC
**Base year cumulative permafrost emissions**					
Base year cumulative carbon, permafrost CO2	4190.01	3.83E+03	4.12E+03	4.62E+03	MtonC
Base year cumulative carbon, permafrost CH4	180.3806	1.75E+02	1.80E+02	1.86E+02	MtonC
**CO2 cycle**					
Percent of CO2 long-term ocean uptake	22.97	4.3	23.0	41.6	%
Percent of CO2 short-term ocean uptake	26.64	23.1	26.6	30.1	%
Percent of CO2 land uptake	26.96	11.4	27.0	42.5	%
Timescale of CO2 long-term ocean uptake	312.54	248.9	312.5	376.2	years

Timescale of CO2 short-term ocean uptake	34.87	25.9	34.9	43.9	years
Timescale of CO2 land uptake	4.26	2.8	4.3	5.7	years
**Sulphate aerosols**					
Sulfate direct (linear) effect in 2015	-0.47	-0.8	-0.4	-0.2	W/m2
Sulfate indirect (log) effect for a doubling of sulphates	-0.23	-0.5	-0.2	0	W/m2
**Sea level rise**					
Sea level rise in 2015	0.19	0.17	0.19	0.21	m
Sea level rise with temperature	1.73	0.7	1.5	3	m/degC
Sea level asymptote	1.00	0.5	1	1.5	m
Time constant of sea level rise (*Gamma*)	386		362		years
**Climate sensitivity**					
Transient climate response	1.77	0.8	1.8	2.7	degC
Feedback response time	28.33	10	20	55	years
Equilibrium warming for a doubling of CO2	2.81	Function of TCR and FRT			degC
**Regional amplification factors**					
EU amplification factor	1.27	1.05	1.23	1.53	
US amplification factor	1.34	1.16	1.32	1.54	
OT amplification factor	1.22	1.14	1.21	1.31	
EE amplification factor	1.65	1.41	1.64	1.9	
CA amplification factor	1.17	1	1.21	1.3	
IA amplification factor	1.01	0.84	1.04	1.15	
AF amplification factor	1.21	0.99	1.22	1.42	
LA amplification factor	1.04	0.9	1.04	1.18	
**SAF emulator**					
Normalised random variable for the emulator	0.00	-1	0	1	dimensionless
**PCF emulator**					
Uncertainty in the initial carbon stock	0	-15	0	15	%
SiBCASA					

Amplification factor for permafrost regions	1.88	1.43	1.88	2.33	dimensionless
Sensitivity for cumulative carbon emissions, CO2	3.19E+04	2.82E+04	3.19E+04	3.57E+04	MtonC/degC
Time lag for cumulative carbon emissions, CO2	61.69	35.49	61.69	87.89	yr
Nonlinear power for cumulative carbon emissions, CO2	0.26	0.11	0.26	0.41	dimensionless
Sensitivity for cumulative carbon emissions, CH4	2.29E+03	1.24E+03	2.29E+03	3.35E+03	MtonC/degC
Time lag for cumulative carbon emissions, CH4	206.29	75.19	206.29	337.38	yr
Nonlinear power for cumulative carbon emissions, CH4	0.25	-0.11	0.25	0.61	dimensionless
JULES					
Amplification factor for permafrost regions	1.94	1.71	1.94	2.16	dimensionless
Sensitivity for cumulative carbon emissions, CO2	6.19E+04	2.47E+04	6.19E+04	9.90E+04	MtonC/degC
Time lag for cumulative carbon emissions, CO2	543.62	252.56	543.62	834.67	yr
Nonlinear power for cumulative carbon emissions, CO2	0.46	-0.23	0.46	1.14	
Permafrost CH4 carbon relative to CO2 carbon	6.12	2.77	6.04	9.53	%
**Impacts of climate change**					
Savings rate	15.00	10	15	20	%
Calibration sea level rise	0.50	0.45	0.5	0.55	m
Calibration temperature	3.00	2.5	3	3.5	degC
Impacts saturate beyond	20.00	15	20	25	%consumption
Statistical value of civilisation	6.1E+10	1.15E+10	5.75E+10	1.15E+11	$M(2015)
Sea level rise					
Sea level initial benefit	0.00	0	0	0	%GDP per m
Sea level impact at calibration sea level rise	1.00	0.5	1	1.5	%GDP
Sea level impact function exponent	0.73	0.5	0.7	1	

Sea level exponent with income	-0.30	-0.4	-0.3	-0.2	
Economic					
Economic impact Burke: temp coeff	-8.30E-03	-1.40E-02	-8.30E-03	-2.62E-03	1/yr per degC
Economic impact Burke: temp squared coeff	-5.00E-04	-6.00E-04	-5.00E-04	-4.00E-04	1/yr per degC^2
Non-economic					
Non-econ initial benefit	0.08	0	0.05	0.2	%GDP per degC
Non-econ impact at calibration temperature	0.63	0.1	0.6	1.2	%GDP
Non-econ impact function exponent	2.17	1.5	2	3	
Non-econ exponent with income	0.00	-0.2	0	0.2	
Discontinuity					
Random variable *(uniform)*	0.5	0	N.A.	1	dimensionless
Tolerable before discontinuity	1.50	1	1.5	2	degC
Chance of discontinuity	20.00	10	20	30	% per degC
Loss if discontinuity occurs	3.00	1	3	5	%GDP
Discontinuity exponent with income	-0.13	-0.3	-0.1	0	
Time constant of discontinuity	20.00	10	20	30	years
Weights (sea level)					
US Sea level weights factor	0.80	0.6	0.8	1	
OT Sea level weights factor	0.80	0.4	0.8	1.2	
EE Sea level weights factor	0.40	0.2	0.4	0.6	
CA Sea level weights factor	0.80	0.4	0.8	1.2	
IA Sea level weights factor	0.80	0.4	0.8	1.2	
AF Sea level weights factor	0.60	0.4	0.6	0.8	
LA Sea level weights factor	0.60	0.4	0.6	0.8	
**Adaptation costs**					

Adaptive costs sea level plateau	0.0233	0.01	0.02	0.04	%GDP per metre
Adaptive costs sea level impact	0.0012	0.0005	0.001	0.002	%GDP per %reduction per metre
Adaptive costs Economic plateau	0.0117	0.005	0.01	0.02	%GDP per degC
Adaptive costs Economic impact	0.0040	0.001	0.003	0.008	%GDP per %reduction per degC
Adaptive costs Non-econ plateau	0.0233	0.01	0.02	0.04	%GDP per degC
Adaptive costs Non-econ impact	0.0057	0.002	0.005	0.01	%GDP per %reduction per degC
Weights (adaptation)					
US Adaptive costs factor	0.80	0.6	0.8	1	
OT Adaptive costs factor	0.80	0.4	0.8	1.2	
EE Adaptive costs factor	0.40	0.2	0.4	0.6	
CA Adaptive costs factor	0.80	0.4	0.8	1.2	
IA Adaptive costs factor	0.80	0.4	0.8	1.2	
AF Adaptive costs factor	0.60	0.4	0.6	0.8	
LA Adaptive costs factor	0.60	0.4	0.6	0.8	
**Mitigation costs**					
CO2					
Uncertainty in BAU emissions in 2100	-22.00	-50	-22	6	% relative to RCP8.5
Initial cutbacks at negative cost	10.00	0	10	20	% of emissions
Initial most negative cost cutback	-100.00	-150	-100	-50	$M(2015) per Mtonne
Initial maximum cutbacks at positive cost	60.00	50	60	70	% of emissions
Initial maximum cutback cost	150.00	100	150	200	$M(2015) per Mtonne
Initial experience stock	150000.00	100000	150000	200000	Mtonne
CH4					
Uncertainty in BAU emissions in 2100	-30.33	-67	-30	6	% relative to RCP8.5
Initial cutbacks at negative cost	10.00	0	10	20	% of emissions
Initial most negative cost cutback	-4983.33	-9200	-4600	-1150	$M(2015) per Mtonne
Initial maximum cutbacks at positive cost	51.67	35	50	70	% of emissions

Initial maximum cutback cost	7283.33	3450	6900	11500	$M(2015) per Mtonne
Initial experience stock	2000.00	1500	2000	2500	Mtonne
N2O					
Uncertainty in BAU emissions in 2100	-7.00	-20	-7	6	% relative to RCP8.5
Initial cutbacks at negative cost	10.00	0	10	20	% of emissions
Initial most negative cost cutback	-8433.33	-17250	-8050	0	$M(2015) per Mtonne
Initial maximum cutbacks at positive cost	51.67	35	50	70	% of emissions
Initial maximum cutback cost	31433.33	2300	23000	69000	$M(2015) per Mtonne
Initial experience stock	53.33	30	50	80	Mtonne
Linear (HGWP gases)					
Uncertainty in BAU emissions in 2100	0.00	-50	0	50	% relative to RCP8.5
Initial cutbacks at negative cost	10.00	0	10	20	% of emissions
Initial most negative cost cutback	-268.33	-460	-230	-115	$M(2015) per Mtonne
Initial maximum cutbacks at positive cost	70.00	60	70	80	% of emissions
Initial maximum cutback cost	383.33	115	345	690	$M(2015) per Mtonne
Initial experience stock	2000.00	1500	2000	2500	Mtonne
Weights (uncertainty in BAU)					
US uncertainty in BAU emissions factor	1.00	0.8	1	1.2	
OT uncertainty in BAU emissions factor	1.00	0.8	1	1.2	
EE uncertainty in BAU emissions factor	1.00	0.65	1	1.35	
CA uncertainty in BAU emissions factor	1.00	0.5	1	1.5	
IA uncertainty in BAU emissions factor	1.00	0.5	1	1.5	
AF uncertainty in BAU emissions factor	1.00	0.5	1	1.5	
LA uncertainty in BAU emissions factor	1.00	0.5	1	1.5	
Weights (uncertainty in negative mitigation costs)					
US negative cost percentage factor	1.08	0.75	1	1.5	

OT negative cost percentage factor	1.00	0.75	1	1.25	
EE negative cost percentage factor	0.70	0.4	0.7	1	
CA negative cost percentage factor	0.70	0.4	0.7	1	
IA negative cost percentage factor	0.70	0.4	0.7	1	
AF negative cost percentage factor	0.70	0.4	0.7	1	
LA negative cost percentage factor	0.70	0.4	0.7	1	
Weights (uncertainty in maximum adaptation costs)					
US maximum cost factor	1.00	0.8	1	1.2	
OT maximum cost factor	1.23	1	1.2	1.5	
EE maximum cost factor	0.70	0.4	0.7	1	
CA maximum cost factor	1.00	0.8	1	1.2	
IA maximum cost factor	1.23	1	1.2	1.5	
AF maximum cost factor	1.23	1	1.2	1.5	
LA maximum cost factor	0.70	0.4	0.7	1	
Evolution in mitigation costs					
Cutbacks at negative cost in 2100 as multiple of 2015	0.88	0.6	0.9	1.15	
Cutbacks at negative cost growth rate	-0.15	Function of the changes in 2100			% per year
Most negative cost in 2100 as multiple of 2015	0.93	0.8	0.9	1.1	
Most negative cost growth rate	-0.08	Function of the changes in 2100			% per year
Maximum cutbacks in 2100 as multiple of 2015	1.12	1	1.1	1.25	
Maximum cutbacks growth rate	0.13	Function of the changes in 2100			% per year
Curvature below zero cost	0.50	0.25	0.45	0.8	
Curvature above zero cost	0.40	0.1	0.4	0.7	
Experience crossover ratio	0.20	0.1	0.2	0.3	
Learning rate (prop. drop in cost for experience doubling)	0.20	0.05	0.2	0.35	proportional drop

Costs in 2100 as multiple of 2015	0.65	0.6	0.65	0.7	
Autonomous technical change	0.51	Function of the changes in 2100			% per year
Equity weights proportion	1.00	1	1	1	

Supplementary Table 18

*Complete list of CMIP5 GCMs used in different parts of the study. The selected models that were used to calibrate the PCF and SAF are marked as **(P)** and **(S)**, respectively.*

Modeling Center (or Group)	Institute ID	Model Name
Commonwealth Scientific and Industrial Research Organization in collaboration with Bureau of Meteorology	CSIRO-BOM	ACCESS1.0<br>ACCESS1.3 **(S)**
Beijing Climate Center, China Meteorological Administration	BCC	BCC-CSM1.1 **(S)**<br>BCC-CSM1.1-m
Beijing Normal University	BNU	BNU-GCM
Canadian Centre for Climate Modelling and Analysis	CCCMA	CanESM2 **(S)**
National Center for Atmospheric Research	NCAR	CCSM4 **(S)**
Community Earth System Model Contributors	NSF-DOE-NCAR	CESM1-BGC<br>CESM1-CAM5 **(S)**
Centro Euro-Mediterraneo sui Cambiamenti Climatici	CMCC	CMCC-CESM<br>CMCC-CM<br>CMCC-CMS
Centre National de Recherches Météorologiques / Centre Européen de Recherche et Formation Avancée en Calcul Scientifique	CNRM-CERFACS	CNRM-CM5 **(P, S)**
Commonwealth Scientific and Industrial Research Organization in collaboration with Queensland Climate Change Centre of Excellence	CSIRO-QCCCE	CSIRO-Mk3.6.0 **(S)**
EC-EARTH consortium	EC-EARTH	EC-EARTH
LASG, Institute of Atmospheric Physics, Chinese Academy of Sciences and CESS, Tsinghua University	LASG-CESS	FGOALS-g2
LASG, Institute of Atmospheric Physics, Chinese Academy of Sciences	LASG-IAP	FGOALS-s2
Geophysical Fluid Dynamics Laboratory	GFDL	GFDL-CM3 **(S)**

**NASA Goddard Institute for Space Studies**	NASA GISS	GISS-E2-H **(P, S)**<br>GISS-E2-H-CC<br>GISS-E2-R<br>GISS-E2-R-CC
**Met Office Hadley Centre (additional HadGEM2-ES realizations contributed by Instituto Nacional de Pesquisas Espaciais)**	MOHC	HadGEM2-ES **(P, S)**
**Institute for Numerical Mathematics**	INM	INM-CM4 **(S)**
**Institut Pierre-Simon Laplace**	IPSL	IPSL-CM5A-LR **(P, S)**<br>IPSL-CM5A-MR<br>IPSL-CM5B-LR
**Japan Agency for Marine-Earth Science and Technology, Atmosphere and Ocean Research Institute (The University of Tokyo), and National Institute for Environmental Studies**	MIROC	MIROC-GCM **(S)**<br>MIROC-GCM-CHEM
**Atmosphere and Ocean Research Institute (The University of Tokyo), National Institute for Environmental Studies, and Japan Agency for Marine-Earth Science and Technology**	MIROC	MIROC5
**Max-Planck-Institut für Meteorologie (Max Planck Institute for Meteorology)**	MPI-M	MPI-GCM-MR<br>MPI-ESM-LR **(P, S)**
**Meteorological Research Institute**	MRI	MRI-CGCM3 **(S)**
**Norwegian Climate Centre**	NCC	NorESM1-M<br>NorESM1-ME **(S)**

Supplementary Table 19

List of CMIP3 GCMs that were used with the JULES LSM to simulate permafrost carbon emissions.

Modeling Center (or Group)	Institute ID	Model Name
**Beijing Climate Center, China Meteorological Administration**	BCC	bccr_bcm2_0
**Canadian Centre for Climate Modelling and Analysis**	CCCMA	cccma_cgcm3_1
**Centre National de Recherches Météorologiques**	CNRM	cnrm_cm3
**Commonwealth Scientific and Industrial Research Organization**	CSIRO	csiro_mk3_0<br>csiro_mk3_5
**Geophysical Fluid Dynamics Laboratory**	GFDL	gfdl_cm2_0<br>gfdl_cm2_1
**NASA Goddard Institute for Space Studies**	NASA GISS	giss_e_h<br>giss_e_r

LASG, Institute of Atmospheric Physics, Chinese Academy of Sciences	LASG-IAP	iap_fgoals1_0_g
European Topic Centre on Climate Change	INGV	ingv_echam4
Institute for Numerical Mathematics	INM	inmcm3_0
Institut Pierre-Simon Laplace	IPSL	ipsl_cm4
Japan Agency for Marine-Earth Science and Technology, Atmosphere and Ocean Research Institute (The University of Tokyo)	MIROC	miroc3_2_hires<br>miroc3_2_medres
Meteorological Institute of the University of Bonn	MIUB	miub_echo_g
Max-Planck-Institut für Meteorologie (Max Planck Institute for Meteorology)	MPI-M	mpi_echam5
Meteorological Research Institute	MRI	mri_cgcm2_3_2a
National Center for Atmospheric Research	NCAR	ncar_ccsm3_0<br>ncar_pcm1
Met Office Hadley Centre	MOHC	ukmo_hadcm3<br>ukmo_hadgem1

Supplementary Note 1

PAGE-ICE v6.22: basic description and system requirements

<u>Authorship, acronyms and key functionalities</u>

PAGE-ICE Nonlinear Arctic Feedbacks	Version	6.22	Date	20/03/2019

Lead author	Dmitry Yumashev	Lancaster University
Contributing author	Chris Hope	University of Cambridge
Original developer of the PAGE model	Chris Hope	University of Cambridge

- PAGE = Policy Analysis of Greenhouse Effect
- ICE = Ice, Climate, Economics
- Nonlinear Arctic Feedbacks: PCF = permafrost carbon feedback; SAF = surface albedo feedback driven by loss of the sea ice and land snow covers (also includes the rest of the world component)

PAGE-ICE is the latest version of the PAGE model as of March 2019. Previous versions: PAGE09, PAGE2002. The model simulates World A, World B and World A-B, allowing one to perform

the following statistical experiments across a wide range of climate and socio-economic scenarios:

- Experiment Family 1: Sensitivity of global climate and economy to nonlinear Arctic feedbacks, PCF and SAF, relative to their legacy values (PCF = 0, SAF = const in line with the 2xCO2 ECS parameter) under a given scenario
- Experiment Family 2: SCCO2 either with the nonlinear or legacy Arctic feedbacks under a given scenario
- Experiment Family 3: Comparison between two scenarios, either with the nonlinear or legacy Arctic feedbacks

System requirements

- Microsoft Windows 7 or higher
- Microsoft Excel 2013 or higher
- @RISK 6.x or higher. Add-in by Palisade allowing one to run statistical (Monte-Carlo) simulations in Excel. Free trial version of @RISK is available at https://www.palisade.com/trials.asp

Description of the individual sheets in the PAGE-ICE workbook

Cockpit	Use the drop down menus to choose the experiment, the underlying climate and socio-economic scenarios, the economic impact function, the weighting and discounting
Results	Statistical post-processing of the key results for the Worlds A, B and A-B
Base data	Climate and socio-economic data for the base year 2015 required to initiate the model, along with the key model settings and the repository of the RCP and SSP scenarios
Library data	Climate and socio-economic parameters of the model with uncertainty. The uncertainty ranges are calibrated according to the latest literature when the data is available, and are expert judgements otherwise. The default probability distributions are subjective in most cases
Policy A, B, and A-B	Chosen mitigation pathways and planned adaptation policies for the Worlds A, B and A-B
Emissions A, B and A-B	Emissions corresponding to the chosen mitigation pathways
Climate A, B and A-B	Future climates in the Worlds A, B and A-B driven by the given emissions pathways and the chosen representations of the Arctic feedbacks (either nonlinear or legacy)
Economy A, B and A-B	Future GDP and population (POP) under the chosen socio-economic pathways, along with the corresponding equity weights and discounting factors
Preventative costs A, B and A-B	Mitigation costs corresponding to the chosen mitigation pathways

Adaptive costs A, B and A-B	Adaptation costs corresponding to the chosen adaptation policy
Impacts A, B and A-B	Impacts due to climate change under the chosen mitigation and adaptation policies. The following impacts categories are included: sea level rise, economic, non-economic, discontinuity
Totals	Summary of the mitigation costs, adaptation costs and climate-driven impacts, and the total economic effect of climate change, for the Worlds A, B and A-B

Glossary

AF	Amplification factor
BAU	Business as usual
CMIP5	Climate models inter-comparison project, phase 5
CMIP3	Climate models inter-comparison project, phase 3
DICE	Dynamic integrated climate-economy (IAM)
ECS	Equilibrium climate sensitivity
FRT	Feedback response time
FUND	Framework for uncertainty, negotiation and distribution (IAM)
GCM	General circulation model (coupled atmosphere, ocean and land)
GHG	Greenhouse gas
GMST	Global mean surface temperature
GWP	Global warming potential
IAM	Integrated assessment model
NDCs	Nationally determined contributions
JULES	Joint UK Land Environment Simulator
LSM	Land surface model
PAGE	Policy analysis of greenhouse effect (IAM)
PAGE-ICE	PAGE – ice, climate and economics
PCF	Permafrost carbon feedback
RCP	Representative concentration pathway
RF	Radiative forcing
SAF	Surface albedo feedback
SiBCASA	Simple Biosphere/Carnegie-Ames-Stanford Approach
SLR	Sea level rise
SPM	Summary for policy-makers
SRES	Special report on emissions scenarios
SSP	Shared socio-economic pathway
TCR	Transient climate response

Overview of the PAGE model

PAGE-ICE v6.22 is based on the earlier versions of the PAGE IAM, PAGE09 and PAGE2002 (Hope, 2006; 2013) and includes a number of updates to climate science and economics in line with the latest literature described in the Methods section of the paper.

The PAGE IAM splits the world into 8 large geopolitical regions: EU, US, other OECD countries (OT), former Soviet Union (EE), China+ (CA), India+ (IA), Africa and Middle East (AF) and Latin America (LA). It estimates climate-driven impacts in each region across four broad categories: sea level rise (coastal flood damage, relocation), economic (both direct and indirect damages to the aggregate economy), non-economic (ecosystems services, public health) and discontinuity (large-scale damages associated with a number of possible tipping points in the climate and economy).[ii] Anthropogenic GHG emissions are split into 6 main classes: CO_2, CH_4, N_2O, linear gases (PFCs, HFC and SF6, sometimes referred to as High GWP gases), sulphate aerosols and the rest of GHGs combined (tropospheric O_3, BC aerosols, OC aerosols, CFCs and HCFCs), and follow IPCC scenarios (SRES or RCPs) extended until year 2200 (2300 in PAGE-ICE). A simple aggregate climate model is used to link the emissions with global temperature rise, which is then scaled for each of the 8 regions and is also used to drive sea level rise as well as the discontinuity impacts.

The rise in regional temperatures serves as a basis for evaluating economic and non-economic impacts, which are calculated as percentage loss/gain of the relevant regional/global GDP in a given future year, and are subtracted from consumption only; endogenous effects on economic growth are not considered in the default model setting. Future GDP and population projections in the 8 world regions follow exogenous scenarios from IPCC (SRES or SSPs). The impacts depend on the increases in the regional temperatures and sea level relative to the corresponding tolerable levels determined by the choice of adaptation spending in each of the 8 regions. The total economic effect of climate change, therefore, consists of mitigation costs (which depend on the levels of ambition in each region under a given emissions scenario), adaptation costs and residual climate impacts. Calculated separately in each region, the total economic effects are equity-weighted depending on the region's relative wealth, and are discounted to the base year (2015 in the default PAGE-ICE setting) using a pure time preference rate and aggregated.

The multiple uncertainties in the global climate and economy are accounted for by performing Monte-Carlo simulations, with over 150 uncertain inputs such as climate sensitivity to CO_2, convexity of the damage functions and discount rates are calibrated using expert climate and economic models. These parameters are summarised below. Due to the limitations of the data, especially when it comes to estimating the impacts of climate change on the economy, most of the input probability distributions are subjective and are approximated by triangular distributions. All the outputs are also in the form of probability distributions.

<u>Discounting</u>

We do not just aggregate the economic impacts over each analysis period in PAGE-ICE, but also discount them in line with a standard methodology adopted from finance, which is common in climate policy assessments based on cost-benefit analysis. The main indicator employed in the analysis is the Net Present Value (NPV) of the economic effect of climate

[ii] PAGE-ICE re-defines the discontinuity impacts sector as being related primarily to the possible large-scale socio-economic effects of climate change such as mass migrations, pandemics and wars, as well as all the other climatic tipping points apart from land permafrost, Arctic sea ice and the two ice sheets.

change and, specifically, of the Arctic feedbacks. It uses the discounting with a pure time preference rate, along with equity weighting based on changes in the marginal utility of consumption with income (Anthoff et al., 2009). As a result, the further in future the impacts are, the less they contribute to the NPV calculation, making the result very different from a simple aggregation. Despite the reduced weight, the impacts that are set to occur in the 22nd and 23rd centuries, i.e. on the timescales associated with the relatively slow climate feedbacks such as carbon emissions from thawing permafrost and sea level rise from melting ice sheets, make an essential contribution to NPV of the total economic effect of climate change.

Monte-Carlo setup and confidence intervals

All the results in the paper are based on 100,000 Monte-Carlo simulations using PAGE-ICE. The corresponding 90% confidence intervals (CIs) for the Monte-Carlo means of the PCF- and SAF-driven changes in the total economic effect of climate change C_{NPV} (net present value until 2300) do not exceed 3% and 3.8% of the respective means under all the scenarios in line with the Paris Agreement. The imaginary Zero Emissions scenario has the relative statistical accuracy of 5% for the mean economic effects of both feedbacks. The 90% CIs of the Monte-Carlo mean effects of the PCF and SAF on C_{NPV} become progressively smaller compared to the means themselves for higher emissions scenarios.

Implementation of the RCP and SSP scenarios and their modifications

RCP scenarios

The RCP database[iii] provides emissions in 5 world regions, while PAGE IAM has 8 regions (see above). For CO_2, CH_4, N_2O and sulphates, we use the following mapping of the RCP regions onto the PAGE regions:

- R5ASIA to CA and IA
- R5LAM to LA
- R5MAF to AF
- R5OECD to EU, US and OT
- R5REF to EE

For the linear gases, we used modifications of the SRES A1B scenario to approximate the linear gases emissions for RCP4.5 and RCP8.5, and assumed that the Kigali Agreement on phasing out HFCs[iv] applies to all regions under RCP2.6.

For the excess forcing, we used modifications of the SRES A1B scenario for tropospheric O_3, BC Aerosols, OC aerosols, CFCs and HCFCs to approximate the excess RF for RCP4.5 and RCP8.5, and adopted the 2016r5low scenario developed by the UK Met Office to provide the excess RF projections for RCP2.6.

[iii] https://tntcat.iiasa.ac.at/RcpDb/dsd?Action=htmlpage&page=welcome
[iv] http://www.unep.org/africa/news/kigali-amendment-montreal-protocol-another-global-commitment-stop-climate-change

The emissions projections for the RCP scenarios are available until 2100. To run PAGE-ICE out to 2300, we assume constant rates of emissions beyond 2100 for all GHGs, which is consistent with the approach used in the selected CMIP5 runs that were extended out to 2300.

The RCP2.6 scenario is based on aggressive early mitigation combined with extensive carbon capture and storage activities, which effectively implies negative net emissions in the latter parts of the 21st century (Van Vuuren et al., 2011).

RCP2.6e scenario

The RCP2.6e scenario follows RCP2.6 with an additional abatement that gives a 50% chance of keeping the GMST increase below either the 1.5°C target or the 2°C target in 2100. The extra abatement is implemented as a ratcheting-up cutback to annual RCP2.6 emissions (same for each of the 6 "policy gases" implemented in PAGE, see above) governed by a compound annual abatement rate, starting in 2020 and running until 2100, which is determined using Risk Optimiser separately for either of the two GMST targets. The marginal abatement cost curve in PAGE-ICE allows one to quantify the very high abatement costs associated both with RCP2.6 and RCP2.6e.

For a given RCP scenario such as RCP2.6, the reductions and/or increases of annual emissions of a selected GHG relative to their base year level E_0 (Mton/yr) can be described by the equation

$$E_t = E_0 \cdot \prod_{t_i=t_0}^{t_i=t} (1 - r_{t_i})$$

where t_0 is the base year, E_t is the projected level of annual emissions in a future year t before 2100, and r_{t_i} are the annual rates of change in future years $t_i \in [t_0, t]$. According to this definition, negative rates r_{t_i} imply emissions increases in a given year.

Introducing a constant extra rate of abatement r_e (per annum) transforms the formula to

$$E_{t,extra} = E_0 \cdot (1 - r_e)^{t-t_0} \cdot \prod_{t_i=t_0}^{t_i=t} (1 + r_{t_i})$$

This formula is implemented for all the "policy gases" in PAGE-ICE (with unique rates for each gas according to the relevant RCP projections) apart from CO_2. CO_2 is the only GHG which reaches negative emissions in the latter parts of the 21st century under the RCP2.6 scenario. The corresponding negative emissions level in 2100 from RCP2.6, denoted as $E_{2100}^{(CO_2)} < 0$, is used to put a constraint on the long-term CO_2 cutback under the RCP2.6e scenario:

$$E_{t,extra}^{(CO_2)} = E_{2100}^{(CO_2)} + \left(E_0^{(CO_2)} - E_{2100}^{(CO_2)}\right) \cdot \left(1 - r_e^{(CO_2)}\right)^{t-t_0} \cdot \prod_{t_i=t_0}^{t_i=t} \left(1 + r_{t_i}^{(CO_2)}\right)$$

These formulae are applicable for t running until 2100. Beyond 2100, annual emission rates for all the GHGs are assumed to be constant:

$$E_t = E_{2100}, \quad t > 2100$$

SSP scenarios

PAGE-ICE uses annual rates of GDP and population change, denoted as $\dot{Y}$ and $\dot{P}$, which are taken from the SSP database[v] and mapped on the 8 PAGE regions. We consider all five SSP scenarios and match them with the relevant RCP scenarios for the emissions according to the RCP-SSP compatibility conditions (Riahi et al., 2017). The more socio-economically unstable and risky SSP3 “regional rivalry” and SSP4 “inequality” scenarios are combined with “middle of the road” SSP2 to define the “medium” SSPM pathway, which is then paired with the medium RCP4.5 emissions scenario. Along with the extreme end pairs SSP1 & RCP2.6 and SSP5 & RCP8.5, the SSPM & RCP4.5 pair provides a plausible development pathway for a word with medium levels of emissions.

The SSP scenarios run until 2100. To extend $\dot{Y}$ out to 2300 while maintaining the trends predicted by the SSP scenarios in the latter parts of the 21st century, we use an exponential extrapolation based on the SSP values in the two final analysis years of the PAGE model that fall within the 21st century: 2075 and 2100. The assumption is that if $\dot{Y}$ is positive in 2100, it subsequently tends to zero exponentially on the long run, while negative $\dot{Y}$ in 2100 is kept constant until 2300. The extrapolation for $t > 2100$ is therefore defined as

$$\dot{Y}_t = \begin{cases} \dot{Y}_{2100} \cdot \exp\left(\frac{t-2100}{\tau_Y}\right), & \tau_Y = (2100-2075) \cdot \left[\ln\left(\frac{\dot{Y}_{2075}}{\dot{Y}_{2100}}\right)\right]^{-1}, \quad \dot{Y}_{2100} > 0 \\ \dot{Y}_{2100}, & \dot{Y}_{2100} < 0 \end{cases}$$

The population growth rates $\dot{P}$, on the other hand, are kept equal to zero beyond 2100 for all the SSP scenarios used.

CO_2 cycle from Joos et al. (2013)

We base the new CO_2 cycle in PAGE-ICE on the latest multi-model assessment of the atmospheric CO_2 response function by Joos et al. (2013). The models used include several CMIP5 GCMs, as well as a number of climate and carbon cycle models of intermediate complexity. All the models were run with the initial pulse of 100 GtC for up to up to $\tau_{max} = 1000$ years. The resulting response function is applicable only on these timescales and has four components:

$$f(\tau) = a_0 + \sum_{n=1}^{3} a_n \cdot \exp\left(-\frac{\tau}{\tau_n}\right), \quad a_0 = 1 - \sum_{n=1}^{3} a_n$$

[v] https://tntcat.iiasa.ac.at/SspDb/dsd?Action=htmlpage&page=about

Supplementary Equation 1

Neither of the components has direct physical meaning, although they could be associated broadly with long-term ocean uptake (a_1, τ_1), short-term ocean uptake (a_2, τ_2) and land uptake (a_3, τ_3) processes. The a_0 terms represents the CO_2 asymptote applicable on the timescales of a millennium (too short for rock weathering to occur). Together, the components of the response function in Supplementary Equation 1 represent one of the best possible empirical fits to the simulations results from each model in the chosen ensemble.

Each model m is characterised its own set of coefficients $\{a_n, \tau_n\}_m$, which are provided in the supplementary materials by Joos et al. We used these coefficients to reconstruct a unique response function $f_m(\tau)$ for each model. The multi-model mean and SD of these functions, denoted as $\mu\big(f_m(\tau)\big)$ and $\sigma\big(f_m(\tau)\big)$, are plotted in Supplementary Figure 18. Introducing a new function $f(\tau)$ using Supplementary Equation 1, with specified uncertainty ranges for all the parameters $\{a_n, \tau_n\}$, we simulated this function multiple times to obtain the corresponding Monte-Carlo mean and SD, denoted as $M\big(f(\tau)\big)$ and $S\big(f(\tau)\big)$. Assuming triangular distributions for $\{a_n, \tau_n\}$, we then ran a statistical optimisation algorithm provided by @Risk Optimiser to find the optimal uncertainty ranges for these distributions that minimise the root mean square misfits simultaneously for the Monte-Carlo mean and SD of the response function relative to the corresponding multi-model mean and SD:

$$\varepsilon_M = \left[\frac{1}{\tau_{max}} \cdot \int_0^{\tau_{max}} \Big(M\big(f(\tau)\big) - \mu\big(f_m(\tau)\big)\Big)^2 \, d\tau\right]^{\frac{1}{2}}$$

$$\varepsilon_S = \left[\frac{1}{\tau_{max}} \cdot \int_0^{\tau_{max}} \Big(S\big(f(\tau)\big) - \sigma\big(f_m(\tau)\big)\Big)^2 \, d\tau\right]^{\frac{1}{2}}$$

The resulting optimal $M\big(f(\tau)\big)$ and $S\big(f(\tau)\big)$ are plotted in Supplementary Figure 18, and the associated uncertainty ranges for $\{a_n, \tau_n\}$ are summarised in Supplementary Table 7. The CO_2 asymptote parameter is expressed through the other exponential weights (Supplementary Equation 1) and therefore does not have its own uncertainty range. The Monte-Carlo mean and SD for a_0 are 23.4% and 10.0%, respectively.

The response function $f(\tau)$ allows one to find the remaining atmospheric CO_2, $C(t)$, in a given year t associated with anthropogenic emissions from the onset of the pre-industrial era, denoted as t_{00}, by taking convolution of the annual anthropogenic emissions $E(t')$ between t_{00} and t:

$$C(t) = \int_{t_{00}}^{t} E(t')\, f(t - t')dt' \equiv \int_{t_{00}}^{t_0} E(t')\, f(t - t')dt' + \int_{t_0}^{t} E(t')\, f(t - t')dt'$$

Supplementary Equation 2

The first term on the right hand side represents the contribution of historic emissions between pre-industrial and base year $t_0 = 2015$, while the second term uses a specified emissions scenario for $E(t)$.

Instead of using historic CO_2 emissions records to evaluate the first component in Supplementary Equation 2, we set $t_{00} = -\infty$ and use the approximation

$$E(t) = E_0 \exp\left(\frac{t - t_0}{\tau_h}\right), \quad t < t_0, \quad \tau_h = \frac{C_0}{E_0}$$

Here $E_0 \approx 41$ $GtCO_2$ per year and $C_0 \approx 2000$ $GtCO_2$ (±10%) are annual and cumulative anthropogenic emissions as of the base year 2015 estimated from the available global records.[vi] The exponential approximation for historic emissions gives the following expression for the remaining atmospheric CO_2 under a given future emissions scenario ($t > t_0$):

$$C(t) = \theta C_0 \cdot \left[a_0 + \sum\nolimits_{n=1}^{3} a_n \cdot \left(\frac{\tau_n}{\tau_h + \tau_n}\right) \cdot \exp\left(\frac{t - t_0}{\tau_n}\right)\right] + \int_{t_0}^{t} E(t')\, f(t - t')dt'$$

Supplementary Equation 3

Here

$$\theta = \left[a_0 + \sum\nolimits_{n=1}^{3} a_n \cdot \left(\frac{\tau_n}{\tau_h + \tau_n}\right)\right]^{-1}$$

is a correction compensating for the exponential approximation of the historic emissions, which ensures that $C(t_0) = C_0$.

For the calculations with the PCF, we add an estimate for the cumulative permafrost CO_2 emissions $C_0^{(p)}$ in the base year obtained from SiBCASA to the relevant anthropogenic cumulative emissions C_0, and then add annual permafrost CO_2 emissions $E^{(p)}(t)$ from the emulator to the annual anthropogenic emissions $E(t)$ under a given scenario.

Supplementary Equation 3 is solved in closed form on each of the analysis periods of PAGE-ICE, separately for the 4 components of the response function, assuming constant anthropogenic emissions during the analysis periods. The solution feeds into the RF formula for CO_2.

PAGE-ICE does not model changes in CO_2 land uptake due to warming and increasing atmospheric CO_2 concentrations (Keenan et al., 2016; Fernandez-Martinez et al., 2019), reductions in CO_2 ocean uptake due to warming and increases in CO_2 ocean uptake driven by carbonate alkalinity changes (Omta et al., 2011), and relies on the default form of the Joos et al. CO_2 response function.

Other updates in the climate science

ECS, TCR and FRT parameters

[vi] Source: http://www.metoffice.gov.uk/research/news/2016/the-global-carbon-budget-2016

The PAGE-ICE calibration of the equilibrium climate sensitivity (ECS, °C) and transient climate response (TCR, °C) parameters follows results from around 30 CMIP5 models, paleo-records and climate models of intermediate complexity featured in the 5th IPCC Assessment Report (IPCC AR5).[vii] Quoting the report:

- 'ECS is likely in the range 1.5°C to 4.5°C (high confidence), extremely unlikely less than 1°C (high confidence), and very unlikely greater than 6°C (medium confidence).'

- 'The TCRs of the 30 AR5 CMIP5 models featured in WGI Table 9.5 vary from 1.1°C to 2.6°C, with a mean of slightly over 1.8°C'.

As in PAGE09, ECS in PAGE-ICE is expressed through TCR and the e-folding feedback response time (FRT, yr) of the upper ocean layers to increased RF:

$$\mathrm{ECS} = \frac{\mathrm{TCR}}{1 - \frac{\mathrm{FRT}}{70} \cdot \left(1 - \exp\left(-\frac{70}{\mathrm{FRT}}\right)\right)}$$

The uncertainty range of FRT is set to be between 10 to 55 years (average of 28 years) to give the Monte-Carlo mean value of ECS of 2.8°C (5-95% range of 1.7°C to 4.2°C). This is consistent with the multi-model mean presented in IPCC AR5, which is based on paleo-records, CMIP5 simulations and 2xCO_2 experiments in climate emulators of intermediate complexity. The calibration is summarised in Supplementary Table 8.

To provide further justification for our ECS parameterisation, we conducted the transient 2xCO_2 and 4xCO_2 and abrupt 4xCO_2 experiments in PAGE-ICE. The results, presented in Supplementary Figure 19 and Supplementary Table 9 are consistent with IPCC AR5 (Tables 9.5 and 9.6, IPCC AR5 WG1).

CMIP5 amplification factors

New probabilistic regional temperature amplification factors (AFs) for the 8 PAGE regions are defined as ratios of the corresponding regional mean surface temperature changes in any given time period to the relevant GMST change, all relative to pre-industrial conditions. The AFs in PAGE-ICE are assumed to be time-independent, and are estimated from CMIP5 multi-model results averaged over the 21st century projections under the four RCP scenarios.

The CMIP5 simulations were driven by time-varying concentrations of GHGs, including aerosols, ozone, as well as GHGs emissions due to land use, volcanic eruptions and solar variability (Taylor et al., 2012). Most models include either semi-interactive or fully interactive aerosol forcing, terrestrial and ocean carbon cycle component and dynamic vegetation, which are key feedbacks of the climate system. In addition, these models include time-evolving ozone field, either prescribed or interactive (Eyring et al., 2013).

[vii] https://www.ipcc.ch/pdf/assessment-report/ar5/wg1/WG1AR5_TS_FINAL.pdf

The CMIP5 simulations were mainly designed to span the pre-industrial period to the end of the 21st century. The historical simulation run from 1850 to 2005, whereas the future simulations cover the 21st century (2006-2100) and follow the RCP scenarios for GHGs concentrations. Not every model in CMIP5 includes future simulations for all the RCP scenarios. Here we use the CMIP5 results available from the British Atmospheric Data Centre.[viii] All simulations are interpolated to a "common" grid of 2.5º by 1.7º latitude/longitude, which amounts to 278 km by 208 km at the equator. The full list of the CMIP5 models used to derive the AFs is given in Supplementary Table 18.

The AF is associated to the phenomenon that changes in the net radiation balance, for example through increasing GHG concentrations, result in larger (or, in some cases, smaller) changes in the mean surface temperature in multiple world regions compared to the global average, which is particularly pronounced in the Arctic (Hansen et al., 1995; Alexeev et al., 2005). In any given region, the amplification factor is defined as:

$$\mathrm{AF}_r = \frac{\Delta \bar{T}_r}{\Delta \bar{T}}$$

Here $\Delta \bar{T}_r$ is the anomaly in the regional mean surface temperature and $\Delta \bar{T}$ is the corresponding anomaly in GMST, calculated relative to the pre-industrial period (1850-1900), and are averaged over 30-year climatological windows.

To estimate the AFs for each of the 8 regions in PAGE-ICE, we use the CMIP5 ensemble during the 21st Century under the four RCPs emission scenarios. Supplementary Figure 20 shows the 30-year running mean time series for the AFs from 2021 to 2085. The variations in the AFs both over time and between different RCP scenarios are small in most regions apart from CA (China and South-East Asia), which justifies the use of time-independent AFs in PAGE-ICE. Supplementary Figure 20 includes boxplots for each individual region to gauge the multi-model mean AFs averaged over the 21st century, separately for each RCP scenario, as well as their associated uncertainties. We use the corresponding multi-scenario boxplots to define the time-independent uncertainty ranges of the AFs in PAGE-ICE, which are given in Supplementary Table 10.

PAGE-ICE generates its own projections for the GMST change T_t relative to pre-industrial conditions in a future analysis year t under a specified emissions scenario using a simple energy balance model (see below). The corresponding regional temperature anomalies relative to pre-industrial, $T_{r,t}$, are then evaluated using the CMIP5-derived AFs from Supplementary Table 10:

$$T_{r,t} = T_t \cdot \mathrm{AF}_r$$

Supplementary Equation 4

[viii] ftp://ftp.ceda.ac.uk/badc/cmip5/data

Equation for GMST based on global energy conservation

PAGE-ICE is different from PAGE09 in the ways both the GMST and regional mean surface temperatures are calculated. It uses global average RF from all the 6 policy gases including sulphates to evaluate the GMST first, and then scales the GMST to the regional temperatures using the AFs. Regional RFs from sulphates are no longer used directly due to the following reasons:

- Climatic response to regional RF is characterised by complex heat exchange mechanisms in the Earth's climate system (Shindell & Faluvegi, 2009). Therefore, it is no longer deemed possible to apply the global ECS parameter to translate regional RF into regional temperature anomalies, as was done in PAGE09;
- The regional temperature scaling based on the AFs already includes the regional RF effects implicitly. Adding the RF from sulphates to the regional temperature equations on top of the AFs would lead to double-counting;
- The new implementation preserves the global energy balance.

The governing equation for the realised GMST, denoted as T, is

$$\frac{dT}{dt} = \frac{1}{\mathrm{FRT}} \cdot \left[T_{eq}(t) - T\right], \quad T_{eq}(t) = \frac{\mathrm{ECS}}{F_{sl} \ln 2} \cdot F^{ant}(t)$$

Supplementary Equation 5

Here $T_{eq}(t)$ is the equilibrium GMST corresponding to a given total anthropogenic RF $F^{ant}(t)$ in analysis year t, which includes globally averaged RFs from all the 6 policy gases in the PAGE model:

$$F^{ant} = F^{CO2} + F^{CH4} + F^{N2O} + F^{Lin} + F^{Sulph} + F^{Exc}$$

F_{sl} (W/m^2) is the RF slope parameter for the logarithmic CO_2 RF law (Hope, 2006), and the ECS and FRT parameters are introduced above

Supplementary Equation 5 is a standard exponential lagged model for the greenhouse effect, which recognises the delay in the upper oceans' warming in response to the RF imbalance. In PAGE-ICE this equation is solved in closed form during each analysis period $t_{i-1} < t < t_i$ using an improved technique based on the following extrapolation for the RF:

$$F^{ant}(t) = F^{ant}_{i-1} + \left(\frac{F^{ant}_{i-1} - F^{ant}_{i-2}}{t_{i-1} - t_{i-2}}\right) \cdot (t - t_{i-1}), \quad t_{i-1} \le t < t_i$$

The resulting closed-form solution of Supplementary Equation 5 is

$$T_i = T_{i-1} + (A_{i-1} - \mathrm{FRT} \cdot B_{i-1} - T_{i-1}) \cdot [1 - \mathrm{EXP}_i] + \Delta t_i \cdot B_{i-1}$$

Supplementary Equation 6

where

$$A_{i-1} = \frac{\text{ECS}}{F_{sl}\ln 2}\cdot F_{i-1}^{ant}, \quad B_{i-1} = \frac{\text{ECS}}{F_{sl}\ln 2}\cdot\left(\frac{F_{i-1}^{ant} - F_{i-2}^{ant}}{t_{i-1} - t_{i-2}}\right),$$

$$\Delta t_i = t_i - t_{i-1}, \quad \text{EXP}_i = \exp\left(-\frac{\Delta t_i}{\text{FRT}}\right)$$

The GMST change T_t in year t described by Supplementary Equation 6 is then scaled to regional mean surface temperature anomalies $T_{r,t}$ using Supplementary Equation 4, which are subsequently used to drive economic and non-economic impacts; the GMST change drives SLR and discontinuity impacts. The global mean land surface temperature anomaly $T_{L,t}$ is given by

$$T_{L,t} = \frac{1}{\text{AREA}_L}\left(\sum\nolimits_r T_{r,t}\cdot\ \text{AREA}_r\right)$$

where $\text{AREA}_L = 148$ million km^2 is the total area of the continents of Earth, equal to the sum the areas AREA_r of all the 8 PAGE regions. The global mean ocean surface temperature anomaly is calculated from T_t and $T_{L,t}$ according to the definition of GMST:

$$T_{O,t} = \frac{T_t\cdot\text{AREA}_E - T_{L,t}\cdot\text{AREA}_L}{\text{AREA}_E - \text{AREA}_L}$$

with $\text{AREA}_E = 510$ million km^2 is the total area of the Earth's surface. PAGE-ICE does not utilise $T_{O,t}$ since the model does not account for marine-specific impacts such as ocean acidification.

Global mean and regional mean surface temperature anomalies in the base year

The realised GMST anomaly in the base year 2015, T_0, is defined according to the estimated average climatology around 2015 relative to pre-industrial conditions (1850-1900), based on the EEA and NOAA temperature records.[ix] T_0 has the mean value of 0.95^{o}C and uncertainty range of ±0.05^{o}C. The realised regional land temperature anomalies in the base year for each region r of PAGE-ICE, $T_{r,0}$, are evaluated using the corresponding realised GMST combined with the regional amplification factors from CMIP5 models introduced in the section above:

$$T_{r,0} = T_0\cdot\text{AF}_r$$

Their area-weighted mean across the entire land area is equal to

$$T_{L,0} = \frac{1}{\text{AREA}_L}\sum\nolimits_r T_{r,0} *\ \text{AREA}_r$$

[ix] EEA: https://www.eea.europa.eu/data-and-maps/indicators/global-and-european-temperature-4/assessment, NOAA: https://www.ncdc.noaa.gov/cag/global/time-series/globe/land_ocean/ytd/12/1880-2016

Initialising the regional temperature anomalies in the base year through the corresponding GMST in the base year and the AFs has a number of advantages:

- A greater consistency with the way the regional temperatures are evaluated in all the analysis years of PAGE-ICE (Supplementary Equation 4);
- A more balanced estimate given greater natural variability in the regional mean surface temperatures compared with the GMST, which makes it harder to come up with credible base year estimates for the regional temperatures using historic records (as was done in PAGE09);
- A reasonable land-to-ocean temperature increase ratio consistent with historic data.

Fat-tailed distribution for sea level rise based on Nauels et al. (2017)

To utilize the explicit representation of sea level rise (SLR) impacts in PAGE and avoid double-counting of the discontinuity-type impacts attributed to the accelerated decline of Greenland and West Antarctica ice sheets, we introduce Gamma distribution for the time constant τ_{SLR} of SLR in the governing equation

$$\frac{d\,\mathrm{SLR}}{dt} = \frac{\mathrm{SLR}_{eq}(T) - \mathrm{SLR}}{\tau_{SLR}}$$

Here

$$\mathrm{SLR}_{eq}(T) = \mathrm{SENS}_{SLR} \cdot T + \mathrm{SLR}_{\infty}$$

is the equilibrium SLR expressed as a linear function of the GMST anomaly; SENS_{SLR} is the sensitivity of SLR to GMST changes, and SLR_{∞} is the asymptotic value corresponding to pre-industrial conditions, which takes into account climatic conditions in the past 2000 years (Grinsted et al., 2010).

We use the latest estimate for global SLR percentiles in 2100 under the SSP5 scenario emissions from Nauels et al. (2017) with extra AIS discharge to calibrate the Gamma distribution parameters for τ_{SLR}. Based on Figure 5 in this reference, the approximate percentile values adjusted to 2015 SLR level are 0.9m (17th), 1.25m (median) and 1.8m (83rd). Running @Risk Optimiser to minimise the root mean square misfit between these percentiles and the corresponding percentiles for the SLR projections in 2100 under RCP8.5 in PAGE-ICE, we find an optimal shape of Gamma distribution for τ_{SLR} plotted in Supplementary Figure 21 with the following parameters:

Mode = 362 years, Mean = 386 years

These values correspond to the 'shape' $k = \mathrm{Mean}/(\mathrm{Mean} - \mathrm{Mode}) = 16$ and 'scale' $\theta = (\mathrm{Mean} - \mathrm{Mode}) = 24$ years in the canonical form. The Gamma distribution for τ_{SLR} is equivalent to a fat-tailed distribution for the relative rate of sea level rise,

$$\lambda_{SLR} = \frac{1}{\tau_{SLR}}$$

with the negative algebraic decay power of $-(1+k) = -17$ at the high-value end (corresponding to rapid SLR response to GMST increases). The Monte-Carlo mean of the resulting SLR in 2100 under the high fossil fuels use scenario (RCP8.5) is 1.57m above pre-industrial levels, which is around 50% higher than the estimate in IPCC AR5 and accounts for recent studies that suggest a possibility of a more rapid collapse of the ice sheets (Golledge et al., 2015; Hansen et al., 2016; Le Bars et al., 2017). The calibration of the SLR parameters in PAGE-ICE is summarised in Supplementary Table 11.

The discontinuity impacts in PAGE-ICE no longer include the risks of catastrophic sea level rise due to the collapse of the ice sheets, and therefore these impacts are downscaled significantly compared with PAGE09 (see below).

Updates in the economics and policy assumptions

Uncertainty in BAU emissions

PAGE-ICE uses RCP8.5 as the high-end emissions scenario which is referred to as BAU in terms of calculating the mitigation costs. However, there is a considerable uncertainty in long-term emissions projections without climate policies, which is in part due to the nature of technological progress in renewable energy. Estimates suggest that the current BAU trajectory is set to reach 4.2°C by 2100,[x] which is less than the 4.8°C predicted for RCP8.5 by CMIP5 simulations (Knutti & Sedláček, 2013). However, a recent study of the upper end of the range of SSP emissions indicates that a pathway exceeding RCP8.5 is still a possibility (Christensen et al., 2018). As a result, the parameter of the PAGE model describing the uncertainty in the long-term BAU projections has been adjusted according to the difference between emissions levels in 2100 under the pathway exceeding RCP8.5 and RCP6.0 scenario.[xi] This is translated to the values of the “Uncertainty in BAU in 2100” parameter for the four main policy gases in PAGE-ICE relative to the RCP8.5 emission levels summarised in Supplementary Table 12.

This scaling is crucial for ensuring that the mitigation costs under all the six climate scenarios considered in the paper are not under- or over-estimated.

Discontinuity impact sector

The discontinuity impact sector in PAGE-ICE accounts only for the possible socio-economic tipping points such as pandemics, mass migration and wars. As a result, the parameters defining the discontinuity impacts have been downscaled considerably compared with PAGE09. The calibration of the discontinuity parameters adopted in PAGE-ICE is given in Supplementary Table 13. These parameters remain highly uncertain due to the difficulty of modelling the impacts of potentially catastrophic socio-economic events, and therefore the discontinuity calibration used in PAGE-ICE ought to be treated as indicative only.

[x] https://www.climateinteractive.org
[xi] See the RCP Database, https://tntcat.iiasa.ac.at/RcpDb/dsd?Action=htmlpage&page=compare

MAC curves and technological learning rates

The marginal abatement cost (MAC) curves in PAGE-ICE are calibrated using the International Energy Agency (IEA) World Energy Outlook study (McKinsey, 2009) together with a more recent review aimed at increasing transparency and comparability in the Paris Agreement in multiple world regions (Aldy et al., 2016). The technological learning rates based on growing experience stock are in line with a recent overview of electricity supply technologies (Rubin et al., 2015). Supplementary Table 14 summarises the PAGE-ICE calibration of the present-day MAC curve and experience-driven technological learning for CO_2, as well as four additional parameters that define the adjustments to the MAC curves for all the GHG expected to occur by 2100 relative to 2015. The latter include changes to emissions cutbacks at negative costs, increases to maximum cutback through measures such as reforestation, and autonomous technological change approximated using autonomous energy efficiency improvements from the IEA Energy Technology Perspectives study (IEA, 2012). This calibration accounts for the technological progress in energy generation, and is applicable across a wide range of mitigation levels, including those at the deep mitigation end, compatible with achieving the 1.5 and 2°C targets from the Paris Agreement.

Economic impact function from Burke et al. (2015)

The PAGE model has four impact sectors: SLR, economic, non-economic and discontinuity. Along with mitigation and adaptation spending, they contribute to the total economic effect of climate change. Compared with PAGE09, PAGE-ICE includes updates to the physical SLR drivers, but not to the economic evaluation of the associated SLR impacts. The discontinuity impacts have been re-defined in line with the updates to the SLR driver and the inclusion of the Arctic land permafrost, land snow and sea ice feedbacks. The now contain only large-scale socio-economic effects of climate change, as well as tipping elements in the climate system other than the nonlinear Arctic feedbacks which are modelled explicitly. The non-economic impacts (gradual changes in ecosystems services and public health not related to the economic output directly) have been updated according to IPCC AR5 (WG2, Chapter 10). We assume that all impacts saturate if they cause GDP losses beyond those seen at the height of the Great Depression in the US in 1933 (around 30% of GDP, or 25% of consumption).[xii]

In addition to all the updates described in the previous sections, the improvements in PAGE-ICE have focused on the economic impacts sector, which represent all the climate-driven effects on the economic output apart from those due to SLR and social discontinuities. The economic impacts are driven by changing mean annual temperatures, and are estimated according to recent macro-econometric analysis of historic temperature shocks on economic growth in multiple countries by Burke et al. (2015), the most comprehensive of its kind to date. We projected the Burke et al. impact function onto the 8 major regions of the PAGE model, and adapted it to fit with the single year consumption-only approach for climate impacts known as level effects, as opposed to growth effects (Piontek et al., 2018). This approach provides a conservative estimate for the climate impacts globally.

[xii] https://www.measuringworth.com/datasets/usgdp/

According to Burke et al., the GDP per capita in a given country with and without the climate effects, denoted respectively as Y and $Y^{(ctrl)}$, changes according to

$$\begin{cases} \frac{d \ln Y}{dt} = \eta(t) + \delta(T(t), T_0) \\ \frac{d \ln Y^{(ctrl)}}{dt} = \eta(t) \end{cases}$$

Supplementary Equation 7

Here $\eta(t)$ is the GDP growth rate as per the chosen SSP scenario, which is determined by the SSP-specific set of assumptions on the socio-economic and technological drivers in the given country, and $\delta(T, T_0)$ is the climate correction to the growth rate, which depends on how much the absolute temperature T in this country, measured in °C, changes relative to its base year value T_0:

$$\delta(T) = h(T) - h(T_0);$$

Here $h(T)$ is the Burke et al. global nonlinear impact function which has the form

$$h(T) = \beta_0 + \beta_1 \cdot T + \beta_2 \cdot T^2 \equiv \gamma_0 + \gamma_1 \cdot (T - T_{cal}) + \gamma_2 \cdot (T - T_{cal})^2$$

$$T_{cal} = 21°\text{C}$$

Supplementary Equation 8

The parameters $\gamma_{1,2,3}$ are all probabilistic, and their values are based on the probability ranges for β_1 and β_2 obtained from the multi-country regression in Burke et al. and listed in their Extended Data Table 1. T_{cal} is the point on the curve with the least uncertainty. The base case estimates for β_1 and β_2, referred to as the "main specification" in Burke et al., correspond to short-term growth effects and are summarised in Supplementary Table 15.

The simulated mean and ±1 SD range of the impact function $h(T)$ described by Supplementary Equation 8 with the values from Supplementary Table 15 are plotted in Supplementary Figure 22. We note that T and T_0 are absolute temperatures in a given country or economic bloc (for example, a region in the PAGE model) and not the anomalies relative to pre-industrial conditions. Also note that although the historic data used by Burke et al. does not have any points beyond $T = 30$°C, we opt to extrapolate the quadratic function to higher temperatures to preserve the pattern of increasing SCCO2 with emissions. As was mentioned earlier, PAGE-ICE has a generic saturation mechanism for the combined impacts of climate change, in which the impacts' limit is set to be of the order of the estimated US GDP loss at the height of the Great Depression. This mechanism is sufficient for capturing the possible saturation of the economic impacts when multiple regional temperatures exceed 30°C (this occurs under high emission scenarios).

Using Supplementary Equation 7, and following Burke et al., we defined the economic impact function as the percentage difference in the GDP in year t_i due to climate change relative to the "control" GDP without climate change:

$$I(t_i) = \frac{Y^{(ctrl)}(t_i) - Y(t_i)}{Y^{(ctrl)}(t_i)} = 1 - \exp\left(\int_{t_i-\Delta t}^{t_i} \delta(T(t'), T_0)\, dt'\right).$$

Supplementary Equation 9

Here Δt is the period prior to t_i during which the climate-driven impacts on economic growth have direct influence on the GDP in year t_i, causing the divergence in the GDP trajectories captured by the impact function. If the integral is negative, the impact function is positive (economic losses), while positive values of the integral imply negative impact function (economic gains).

Burke et al. set $\Delta t = t_i - t_0$, therefore assuming that the integral in Supplementary Equation 9 is taken from t_0 to t_i, i.e. the "memory effects" of climate impacts on economic growth go back to the base year. This interpretation is in line with the shot-term growth effects (but not persistent growth effects, which cause even larger long-term GDP losses). However, after performing lagged regressions for up to 5 years, Burke et al. concluded that: "while we can clearly demonstrate that there is a nonlinear effect of temperature on economic production, we cannot reject the hypothesis that this effect is a true growth effects nor can we reject the hypothesis that it is a temporary level effect" (Supplementary Materials, p15).

Our understanding is that a given country could experience either the level effects, short-term growth effects or persistent growth effects associated with climate impacts on economy, as per the distinction in Burke et al. (Extended Data, Fig. 2a). Moreover, the nature of response to climate stressors may even switch between the three different options over time. This will depend on each country's unique set of socio-economic policies, as well as global economic trends, which are set to determine its vulnerability and resilience to climate impacts.

In PAGE-ICE, however, we use a more conservative assumption that all the climate-driven losses (gains) are fully repaired (spent on consumption) in the end of each year, which corresponds to the level effects. This implies that $\Delta t = 1$ in Supplementary Equation 9, and therefore the impacts do not propagate beyond the year during which they occur, giving the following impact function:

$$I(t_i) = 1 - \exp\left(\int_{t_i-1}^{t_i} \delta(T(t'), T_0)\, dt'\right).$$

Supplementary Equation 10

The implication is that in the end of each year the economy rebounds to the same trajectory it was on prior to the losses (gains) occurring in that year. This is in line with the default approach to climate impacts in the PAGE model, which is based on level effects rather than growth effects, and provides an incremental change in the modelling framework, also allowing one to compare directly with the PAGE09 default impact function.

Most importantly, our preference towards the level effects approach is driven by the desire to be able to provide the lowest possible estimate for the impacts of climate change that is

unlikely to be reduced further, regardless of the nature of the individual countries' response to future climate stressors.

The base-year absolute temperatures in each of the 8 PAGE regions (Supplementary Table 16), required for initialising the Burke et al. function, are obtained from Era-Interim re-analysis between 1979 and 2005,[xiii] and are weighted over population count in each cell.[xiv] They are adjusted to the PAGE base year climatology using the EEA and NOAA temperature records (see above).

Complete list of the uncertain parameters in PAGE-ICE

Supplementary Table 17 provides complete list of the uncertain parameters in PAGE-ICE. Unless otherwise stated, all the parameters are approximated by triangular distributions defined by the min, mod and max values stated in the table. A small number of parameters in Supplementary Table 17 such the 2xCO2 equilibrium climate sensitivity (ECS) or the autonomous technological progress rate are expressed as functions of one or more "primary" parameters with triangular distributions. The time constant of sea level rise is approximate by Gamma distribution. Further comments are provided in the Excel workbook of PAGE-ICE.

Supplementary Note 2

Using SiBCASA and JULES as representative LSMs to simulate permafrost carbon emissions

SiBCASA and JULES are contrasting land surface models (LSMs) which are able to trace the fate of old (frozen) permafrost carbon under a specified future climate scenario. The two LSMs adopt different modelling philosophies to account for a number of highly uncertain biophysical processes in thawing permafrost soils. Supplementary Figure 1 shows the cumulative amount of old permafrost carbon emitted into the atmosphere in the form of CO_2 for the SiBCASA and JULES simulations with multiple GCMs under RCP8.5, plotted against the previously published values (Schaefer et al., 2014; Schuur et al., 2015). JULES appears to be on the lower end and SiBCASA on the upper end of the reference multi-model studies, and the two models' responses to the warming have contrasting dynamics. In particular, JULES seems to underperform in 2100 while SiBCASA appears to over-perform in 2300.

The primary underlying cause of the differences between SiBCASA and JULES is the scaling of respiration with depth, which is applied in JULES to account for the factors that are currently missing from the model such as anoxia, priming effects, soil mineral surface and aggregate stabilization. The scaling factor implies that the respiration of the permafrost carbon is inhibited compared to the carbon in the active layer. SiBCASA, which models anoxia but

xiii https://www.ecmwf.int/en/forecasts/datasets/archive-datasets/reanalysis-datasets/era-interim

xiv Gridded population map from http://sedac.ciesin.columbia.edu/data/set/gpw-v4-population-count-rev10/data-download

excludes the other factors listed above, does not have the vertical scaling of respiration. As a result, the two LSMs have very different turnover times of the soil carbon. The current state of permafrost observations does not allow one to choose one approach in favour of the other. By working with the SiBCASA and JULES pair we are able to capture the essential uncertainties in state-of-the-art permafrost modelling.

The contrast between the SiBCASA and JULES dynamics is also evident from the fact that the statistical emulator of the permafrost emissions developed in this study, which captures the essential features of these two models' simulations, produces very different parameter values when calibrated to either of the two models. In particular, the magnitude of the characteristic lag between the warming and the permafrost emissions, as well as the nature of its dependence on the "distance" from the equilibrium, vary considerably between the two models. Further details are provided in the sections below.

Permafrost amplification factor

The amplification factor (AF) is defined based on the mean annual surface air temperatures averaged across the estimated permafrost regions during pre-industrial (1850-1900) conditions. Supplementary Figure 5 illustrates the permafrost AFs simulated by the five CMIP5 models used in SiBCASA runs under RCP8.5 and RCP4.5 out to 2300 (see Supplementary Table 1 below for the full description of the CMIP5 models). The strong linear relationship between the GMST anomaly and the average permafrost temperature anomaly, with the average slope of 1.8 (±0.3), suggests that the permafrost AF is nearly constant within the simulated range of GMST projections (GMST anomalies of up to 12°C above pre-industrial conditions).

Calibration algorithm for the PCF emulator

The dynamic emulator of the permafrost carbon emissions is based on a nonlinear first order ODE introduced in the Methods:

$$\frac{dC}{dt} = \frac{C_{max}}{\tau\, \varphi_\tau(T)} \cdot \left(\frac{\max\left(C_{eq}(T) - C, 0\right)}{C_{max}} \right)^{(1+p)\, \varphi_p(T)}$$

Supplementary Equation 11

We calibrated the parameters of Supplementary Equation 11 separately for the CO_2 and methane fluxes simulated by SiBCASA, and for the CO_2 fluxes simulated by JULES. The simulations of these two LSMs used gridded daily CMIP5 (SiBCASA) or CMIP3 (JULES) projections for temperature and precipitation in the permafrost region, which was defined according to the simulated pre-industrial permafrost extent (1850-1900). The emulator was forced by mean annual GMST projections from the same CMIP5 experiments throughout the fitting procedure. In the emulator runs, the gridded GMST projections from the CMIP5 models were converted into mean annual temperatures T averaged across the pre-industrial permafrost region using the corresponding amplification factors derived separately for each CMIP5 or CMIP3 model:

$$T = \mathrm{AF_p} \cdot \mathrm{GMST}.$$

The emulator calibration involved applying a standard gradient-free "fminsearch" algorithm in MATLAB that minimises a normalised misfit between the LSM simulations and a numerical solution of Supplementary Equation 14 defining the emulator (below) by adjusting three main parameters: ω, τ, p. The normalised misfit, $\varepsilon_{m,s}$, is defined as

$$\varepsilon_{m,s} = \frac{1}{\max\limits_{m',s'}\left(C^{LSM}_{m',s'}(t_N)\right)} \cdot \left[\frac{1}{N}\sum_{i=1}^{N}\left(C_{m,s}(t_i) - C^{LSM}_{m,s}(t_i)\right)^2\right]^{\frac{1}{2}},$$

Supplementary Equation 12

where t_i is time, running from 2000 to 2300; $N = 300$ is the number of time steps; $C^{LSM}_{m,s}(t_i)$ is the cumulative carbon flux at time t_i from the LSM simulations (SiBCASA or JULES) with the GCM m under the scenario s; and $C_{m,s}(t_i)$ is the corresponding numerical solution of Supplementary Equation 14.

Each combination of a GCM and scenario in the LSM simulations produces its own set of optimal parameters $(\omega, \tau, p)_{m,s}$ that minimises $\varepsilon_{m,s}$. It turns out that such a procedure yields inter-scenario biases in the parameters ω, τ, p, where the parameters appear to cluster around different values depending on the scenario. To reduce the biases, we introduced the nonlinear corrections φ_ω, φ_τ, φ_p, which are functions of the permafrost temperature. These were obtained from an iteration algorithm assuming that the optimal parameters $(\omega, \tau, p)_{m,s}$ on each iteration are functions of the relevant permafrost temperatures $T_{m,s}(t_N)$ in year $t_N = 2300$ (end of the timespan for the datasets). The functional forms used for the temperature corrections are:

$$\varphi_{\omega,\tau}(T) = \begin{cases} 1 + \delta_{\omega,\tau} \cdot \left(\dfrac{T - 0.5 \cdot T_{max}}{T_{max}}\right), & \text{SiBCASA CO2} \\ \left(\dfrac{T}{0.5 \cdot T_{max}}\right)^{\delta_{\omega,\tau}}, & \text{SiBCASA CH4, JULES CO2} \end{cases}$$

$$\varphi_p(T) = 1 + \delta_p \cdot \left(\frac{T - 0.5 \cdot T_{max}}{T_{max}}\right), \quad \text{all cases}$$

Supplementary Equation 13

Here δ_ω, δ_τ, δ_p are constant dimensionless slopes adjusted on each iteration to reduce the inter-scenario bias, and T_{max} is the highest permafrost temperature anomaly from pre-industrial achieved in the GCM simulations with either SiBCASA or JULES. The adjustments to the slopes were performed by means of an appropriate polynomial fitting between $(\omega, \tau, p)_{m,s}$ and $T_{m,s}(t_N)$. The nonlinear correction δ_ω, for example, is adjusted as follows between two consecutive iterations:

$$\delta_\omega^{(iter)} = \delta_\omega^{(iter-1)} + \mu_\omega \cdot s_\omega^{(iter-1)}, \quad s_\omega^{(iter-1)} = \mathrm{slope}\left\{\omega_{m,s}^{(iter-1)}, T_{m,s}(t_N)\right\}$$

Here slope{·} is the slope parameter for either a linear (SiBCASA CO_2; $\omega \sim T$) or log-linear (SiBCASA methane and JULES CO_2; $\ln\omega \sim \ln T$) polynomial fitting of the set of values $\omega_{m,s}$ to the set of values $T_{m,s}(t_N)$ across all the models m and scenarios s, and μ_ω is an empirically determined damping factor that makes the iterations converge.[xv] For SiBCASA CO_2, we have the following expression for the slope parameter $s_\omega^{(iter)}$:

$$\left\{\frac{\omega_{m,s}^{(iter)}}{\mathrm{mean}_{m',s'}\left(\omega_{m',s'}^{(iter)}\right)}\right\} = s_\omega^{(iter)} \cdot \left\{\frac{T_{m,s}(t_N) - 0.5 \cdot T_{max}}{T_{max}}\right\} + \text{fitting error},$$

For SiBCASA methane and JULES CO_2, this is modified to

$$\ln\left\{\frac{\omega_{m,s}^{(iter)}}{\mathrm{mean}_{m',s'}\left(\omega_{m',s'}^{(iter)}\right)}\right\} = s_\omega^{(iter)} \cdot \ln\left\{\frac{T_{m,s}(t_N)}{0.5 \cdot T_{max}}\right\} + \text{fitting error},$$

The iterative adjustments to δ_τ and δ_p follow the same procedure, depending on which functional form is used according to Supplementary Equation 13.[xvi]

The iterations are stopped when each of the relevant correlation coefficients,

$$R^2\{\omega_{m,s}, T_{m,s}(t_N)\}, \quad R^2\{\tau_{m,s}, T_{m,s}(t_N)\}, \quad R^2\{p_{m,s}, T_{m,s}(t_N)\},$$

falls below a required minimum threshold of 0.01,[xvii] ensuring that the optimal parameter sets $(\omega, \tau, p)_{m,s}$ are quasi-independent from the scenarios or models that were used to obtain them. The latter allows us to use these sets of values to construct the corresponding probability distributions for ω, τ, p in PAGE-ICE, which are expected to work throughout the simulated range of temperatures. This clear statistical criterion implies that our PCF emulator is robust and its range of applicability can be extended to scenarios such as the 1.5°C and 2°C targets (Supplementary Figure 9 in the main article). Further technical details of the fitting algorithm and the resulting numerical values are given in the sections below.

CO_2 component of the emulator, SiBCASA

Supplementary Figure 6 shows projected cumulative permafrost CO_2 emissions until 2300 under the RCP4.5 and RCP8.5 scenarios, generated by SiBCASA and by our model emulator, individually for each of the five CMIP5 models employed in the SiBCASA simulations. The plots in Supplementary Figure 6 demonstrate a very good fitting accuracy.

Supplementary Figure 7 shows the time-constant fitting parameters $(\omega, \tau, p)_{m,s}$ for the CO_2 component of the PCF emulator for all the models m and scenarios s, plotted against the

[xv] $\mu_\omega = 0.5$ SiBCASA CO_2; $\mu_\omega = 0.25$ for SiBCASA methane and JULES CO_2 emulator fitting.
[xvi] $\mu_\tau = 0.5$, $\mu_p = 0.1$ SiBCASA CO_2; $\mu_\tau = \mu_p = 0.25$ for SiBCASA methane and JULES CO_2 emulator fitting.
[xvii] Established empirically to allow for convergence of the iterations to calibrate the emulator, separately, to the SiBCASA CO_2, SiBCASA methane and JULES CO_2 simulations.

relevant permafrost temperature projections $T_{end} = T_{m,s}(t_N)$ in year $t_N = 2300$ (end of the timespan for the datasets). The values $(\omega, \tau, p)_{m,s}$ are obtained through minimising the normalised misfit between the SiBCASA and emulator projections for the cumulative carbon emissions plotted in Supplementary Figure 6. They are adjusted further in a special iteration algorithm that minimises the inter-scenario bias, as is described in the section above. The residual correlations between $(\omega, \tau, p)_{m,s}$ and $T_{m,s}(t_N)$ are close to zero.

Supplementary Figure 8 shows how the correlation coefficients and the corresponding slopes δ_ω, δ_τ, δ_p, which define the temperature-dependent corrections φ_ω, φ_τ, φ_p to the parameters ω, τ, p, evolve throughout the iterations. The slopes appear to converge to constant values summarised in Supplementary Table 2, together with the residual correlations. The corresponding probability distributions defined by the sets of values $(\omega, \tau, p)_{m,s}$ from the final iteration are given in Supplementary Table 5.

Supplementary Figure 9 illustrates how the maximum normalised misfit, $\max(\varepsilon_{m,s})$, evaluated across all the models m and scenarios s using Supplementary Equation 12, changes throughout the iterations. The overall tendency is for the maximum misfit to decrease with iterations before converging to the value of around 1.5%. Therefore, the algorithm achives both low misfits between the emulator and the SiBCASA simulations across all the models and scenarios considered, and low levels of the residual inter-scenario bias for each of the three emulator parameters $(\omega, \tau, p)_{m,s}$. This implies that our PCF emulator is robust and its range of applicability can be extended to scenarios such as the 1.5°C and 2°C targets.

<u>Methane component of the emulator, SiBCASA</u>

The nonlinear corrections φ_ω, φ_τ, φ_p for the methane component of the PCF emulator of the SiBCASA simulations have a different structure compared with the CO_2 SiBCASA component (Supplementary Equation 13). The fitting results are presented in the Supplementary Figure 10, Supplementary Figure 11, Supplementary Figure 12, Supplementary Figure 13 and Supplementary Table 3.

<u>CO2 component of the emulator, JULES</u>

The emulator of the JULES CO_2 simulations has the same structure as the emulator for the methane component of the SiBCASA simulations (Supplementary Equation 13), although the resulting numerical values differ. JULES was run with 22 CMIP3 GCMs under 3 emissions scenarios each, giving 66 samples for establishing the statistics in the emulator parameters $(\omega, \tau, p)_{m,s}$. In comparison, SiBCASA was run with only five CMIP5 GCMs under 2 scenarios, giving 10 samples to derive the emulator statistics. The fitting results for the emulator of the JULES CO_2 simulations are presented in Supplementary Figure 14, Supplementary Figure 15, Supplementary Figure 16, Supplementary Figure 17 and Supplementary Table 4.

Uncertainty ranges and numerical scheme for the PCF emulator in PAGE-ICE

Supplementary Table 5 summarises the values of the uncertain parameters defining the PCF emulator, separately for the CO_2 and methane fluxes simulated by SiBCASA. Supplementary

Table 6 replicates the same for the emulator of the CO_2 fluxes simulated by JULES. All the values were obtained using the iterative fitting algorithm described in the sections above. The uncertainty ranges are used to define the associated probability distributions in PAGE-ICE.

The mean equilibrium carbon sensitivity in the CO_2 JULES emulator is around twice larger than in the CO_2 SiBCASA emulator, which in turn is roughly 15 times larger than for the methane SiBCASA emulator. The characteristic time lag in the CO_2 JULES emulator is also the highest, and is nearly 10 times bigger than the lag for the CO_2 SiBCASA emulator. The power parameters determine the nature of convergence to the equilibrium, and they are broadly similar across all the components, albeit with different uncertainty ranges. A combination of these factors implies that the permafrost carbon emissions described by the governing Supplementary Equation 14 of the emulator are the highest for the CO_2 SiBCASA component. The timing of the emissions also varies greatly between the components, as is illustrated in Supplementary Figure 6, Supplementary Figure 10 and Supplementary Figure 14.

In total, each of the three emulator components (CO_2 SiBCASA, methane SiBCASA, CO_2 JULES) has 4 statistical parameters (Supplementary Table 5 and Supplementary Table 6). These are complemented by the 3 slope parameters δ_ω, δ_τ, δ_p defining the nonlinear corrections for the emulator parameters, given in Supplementary Table 2, Supplementary Table 3 and Supplementary Table 4. These parameters are derived by analysing inter-model and inter-scenario datasets and are deterministic as a result.

We solve the governing equation for the cumulative carbon emissions

$$\frac{dC}{dt} = \frac{C_{max}}{\tau\,\varphi_\tau(T)} \cdot \left(\frac{\max\left(C_{eq}(T) - C, 0\right)}{C_{max}}\right)^{(1+p)\,\varphi_p(T)}$$

$$C_{eq}(T) = \min(\omega\,\varphi_\omega(T) \cdot T, C_{max})$$

Supplementary Equation 14

in closed form on each analysis period $t_{i-1} < t < t_i$, which is possible since the temperature is assumed to be constant during each period. Defining

$$q(T_i) = 1 - (1+p) \cdot \varphi_p(T_i),$$

the resulting numerical scheme is:

$$C_i = \begin{cases} C_{i-1}, & C_{eq}(T_i) \le C_{i-1} \\ C_{eq}(T_i), & \left(\frac{C_{eq}(T_i) - C_{i-1}}{C_{max}}\right)^{q(T_i)} \le \frac{q(T_i)}{\tau\,\varphi_\tau(T_i)} \cdot (t_i - t_{i-1}) \\ C_{eq}(T_i) - C_{max} \cdot \left[\left(\frac{C_{eq}(T_i) - C_{i-1}}{C_{max}}\right)^{q(T_i)} - \frac{q(T_i)}{\tau\,\varphi_\tau(T_i)} \cdot (t_i - t_{i-1})\right]^{\frac{1}{q(T_i)}}, & \text{otherwise} \end{cases}$$

Supplementary Equation 15

Once the permafrost carbon emissions based on SiBCASA and JULES emulators are computed in each analysis year using Supplementary Equation 15 (either CO_2 or methane components), they are added together with equal weights, and then multiplied by the uncertainty factor for the initial permafrost carbon stock (Hugelius et al., 2014):

$$C(t) = 0.5 \cdot \left(C^{(SiB)}(t) + C^{(JUL)}(t)\right) \cdot (1 + \chi), \quad \chi = 0.01 \cdot \mathrm{Triang}(-15, 0, 15).$$

Supplementary Equation 16

As JULES does not model permafrost methane emissions explicitly, the latter were inferred from its CO_2 emissions using observational constraints (Schädel et al., 2016):

$$C_{CH4}^{(JUL)} = C_{CO2}^{(JUL)} \cdot \theta, \quad \theta = 0.01 \cdot \mathrm{Triang}(2.8, 6, 9.5).$$

The total cumulative permafrost carbon emissions $C(t)$ from Supplementary Equation 16 (either CO_2 or methane components) were added to the carbon cycle of the PAGE-ICE model to estimate the effects of the PCF.

Supplementary Note 3

Details of the CMIP5 models used for the SAF emulator

We computed the SAF from 16 CMIP5 models that have the necessary variables to apply Winton's ALL/CLR method: rsus, rsds, rsdscs and rsdt (Winton, 2005; 2006a). A complete list of these models can be found in Supplementary Table 1. The following eight models provided the variables until 2300: BCC-CSM1.1, CCSM4, CNRM-CM5, CSIRO-Mk3.6.0, GISS-E2-H, HadGEM2-ES, IPSL-CM5A-LR and MPI-ESM-LR. The other eight models provided the data until 2100. Each model has its own domains for Arctic sea-ice (sic) and land snow (snc) covers based on their respective monthly maximum extents during the pre-industrial period (1850-1900), with the exception of the model IPSL-CM5A-LR, which only allowed the computation of a northern hemispheric sea-ice domain. We did not apply bias-correcting to the sea ice, land snow and GMST simulations to preserve the internal consistency in the physics for each model.

Fixed pre-industrial cloud test

Winton's ALL/CLR method to compute the SAF uses a parameterization for upward atmospheric reflectivity (Winton 2006a, Eq. 4). It depends on the relation between downward shortwave fluxes at the surface under all skies (with clouds) and clear skies (assumed to be no clouds). According to Winton (2006a), the "reflectivity is mainly due to reflections from the undersides of clouds and will depend upon cloud parameters such as cloud water path and effective drop radius", while "the coefficients in Eq. (4) are round numbers based on physical reasoning and are not fit to any particular model result (Winton, 2005)." Therefore, Winton's

ALL/CLR method includes the effect of clouds on the value of the SAF. However, the method does not distinguish between changes in the cloud cover (cloudiness) in warmer climates (Collins et al., 2013) and the localised changes in the clouds driven specifically by changes in the surface albedo, which were isolated using radiative kernels (Soden et al., 2008).

The effect of clouds on the SAF in the Winton's ALL/CLR method can be demonstrated in a simple experiment. The method allows one to compute the change in shortwave radiation flux (W/m^2) as the difference between a reference period and a perturbation experiment (Winton 2006a, Eq. 6). This difference depends on both the reference and perturbed values of the upward and downward shortwave radiation, surface albedo and atmospheric reflectivity. As mentioned above, the atmospheric reflectivity includes the effect of clouds. We conducted a simple experiment where the atmospheric reflectivity always represents the reference period (which in our case is the 1850-1900 baseline pre-industrial climatology) and thus the cloudiness remains constant, while the other variables change during the perturbation experiment (30-year climatological windows moving from the reference period until 2100 or 2300 under RCP8.5). We refer to this as the "fixed pre-industrial cloud" experiment. The effect of time-constant cloud cover fixed at the pre-industrial climatology is to increase the SAF for all of its three main components considered in the study (sea ice, land snow and rest of the world), which is illustrated in Supplementary Figure 2. This result could be interpreted as follows. An increased global cloudiness for transient climate simulations under RCP8.5 (Collins et al., 2013) reflects more shortwave radiation back into the atmosphere compared with the cloud cover from the pre-industrial period, which leads to a small reduction in the actual SAF relative to its hypothetical value computed with the fixed pre-industrial cloud cover.

As mentioned earlier, the fixed pre-industrial cloud experiment cannot tell us whether the dampening effect of the clouds on the sea ice and land snow SAF components is associated specifically with the localised changes to the surface albedo (Soden et al., 2008; Caldwell et al., 2016), or whether it is mainly driven by the overall global changes to cloudiness in warmer climates. Since clouds are part of the fully coupled CMIP5 climate models, both of these effects are implicitly included in the diagnostic shortwave variables used in the ALL/CLR method of calculating the SAF by Winton that we adopted here.

Implementation of the SAF emulator in PAGE-ICE

The statistical emulator of the nonlinear SAF computed using Winton's ALL/CLR method based on CMIP5 simulations is described in the main article (Methods). The emulator recognises that the SAF is implicitly included in the $2xCO_2$ equilibrium climate sensitivity parameter (ECS), which is central to modelling the greenhouse effect in IAMs like PAGE, DICE and FUND.[xviii] Without acknowledging the baseline level of the SAF used in the ECS parameter, which we refer to as the legacy value, simply adding it to the anthropogenic RF would amount to double-counting. To date, none of the IAMs have had a temperature-varying ECS to reflect the nonlinear (state-dependent) nature of planetary feedbacks such as the SAF. The $2xCO_2$ ECS parameter in PAGE-ICE is consistent with the range in IPCC AR5, which is based on paleo-records, CMIP5 simulations and $2xCO_2$ experiments in climate emulators of intermediate

[xviii] Unlike the CMIP5 definition of the ECS, which is based on the abrupt $4xCO_2$ experiment, PAGE-ICE employs the more generic IPCC AR5 definition based on the $2xCO_2$ increase relative to pre-industrial conditions.

complexity. The corresponding mean equilibrium warming is 2.8°C (5-95% range of 1.7°C to 4.2°C). According to the GCMs' simulations analysed, the statistical mean value of the average level of the global SAF for the period between pre-industrial conditions and the 2xCO_2 ECS warming is $f^{(ecs)} = 0.349 \pm 0.045$ W/m^2/K, which is in good agreement with historic data (Flanner et al., 2011; Pistone et al., 2014; Cao et al., 2015). The implicit baseline assumption in IAMs to date has been that of a constant legacy SAF equal to $f^{(ecs)}$. This is equivalent to the RF due to the surface albedo changes extrapolated linearly with the GMST anomaly T relative to the pre-industrial conditions (1850-1900):

$$F^{(ecs)}(T) = f^{(ecs)} \cdot T.$$

Supplementary Equation 17

To capture the effect of the state-dependent SAF under future climate scenarios, we used the nonlinear emulator (Methods) and evaluated it in the analysis years t_i of PAGE-ICE. We employed piece-wise linear interpolation with respect to GMST for each analysis period $t_{i-1} < t < t_i$ characterized by the temperature range $T_{i-1} < T < T_i$, resulting the following SAF-driven increase in the RF relative to the pre-industrial (1850-1900) conditions:

$$F^{(int)}(T) = F(T_{i-1}) + f_{i-1}^{(int)} \cdot (T - T_{i-1}), \quad f_{i-1}^{(int)} = \frac{F(\hat{T}_i) - F(T_{i-1})}{\hat{T}_i - T_{i-1}}.$$

Supplementary Equation 18

Here $\hat{T}_i$ is a preliminary temperature estimate in year t_i before the nonlinear SAF correction is introduced (that is, based on the legacy SAF value introduced above), $F(T)$ is the probabilistic RF from the SAF emulator which is given in the Methods section of the main article and Equation 2 therein, $f_{i-1}^{(int)}$ is the resulting constant SAF approximation over the analysis period $t_{i-1} < t < t_i$, and $F^{(int)}(T)$ is the corresponding linearly interpolated RF for the same analysis period.

The difference between the nonlinear RF (Supplementary Equation 18, interpolated over a given analysis period) and linearly extrapolated cumulative RF (Supplementary Equation 17, corresponding to the constant legacy SAF) during the analysis period $t_{i-1} < t < t_i$ is

$$\Delta F^{(corr)}(T) = F^{(int)}(T) - F^{(ecs)}(T) = \left(F(T_{i-1}) - f_{i-1}^{(int)} \cdot T_{i-1}\right) + \left(f_{i-1}^{(int)} - f^{(ecs)}\right) \cdot T$$
$$\equiv \Delta F_{i-1}^{(corr)} + \Delta f_{i-1}^{(corr)} \cdot T, \qquad T_{i-1} < T < T_i$$

where we defined

$$\Delta F_{i-1}^{(corr)} = F(T_{i-1}) - f_{i-1}^{(int)} \cdot T_{i-1}, \qquad \Delta f_{i-1}^{(corr)} = \left(f_{i-1}^{(int)} - f^{(ecs)}\right).$$

As a result, the nonlinear correction to the SAF alters the governing equation for the GMST change in PAGE-ICE by adding extra terms to the total anthropogenic RF, $F^{(ant)}(t)$, which modifies the 2xCO_2 equilibrium climate sensitivity parameter ECS (°C) and the e-folding

feedback response time FRT (yr) of the upper ocean layers to increased RF. For the analysis period $t_{i-1} < t < t_i$, the GMST equation becomes:

$$\frac{dT}{dt} = \frac{1}{\mathrm{FRT}_{i-1}^{(corr)}} \cdot \left[\frac{\mathrm{ECS}_{i-1}^{(corr)}}{F_{sl} \ln 2} \cdot \left(F^{(ant)}(t) + \Delta F_{i-1}^{(corr)} \right) - T \right].$$

Supplementary Equation 19

Here

$$\mathrm{ECS}_{i-1}^{(corr)} = \mathrm{ECS} \cdot \left[1 - \frac{\mathrm{ECS}}{F_{sl} \ln 2} \Delta f_{i-1}^{(corr)} \right]^{-1}, \qquad \mathrm{FRT}_{i-1}^{(corr)} = \mathrm{FRT} \cdot \left[1 - \frac{\mathrm{ECS}}{F_{sl} \ln 2} \Delta f_{i-1}^{(corr)} \right]^{-1}$$

are the modified ECS and FRT parameters adjusted in each analysis year according to the change $\Delta f_{i-1}^{(corr)}$ in the SAF relative to the constant legacy value; F_{sl} (W/m^2) is the RF slope parameter for the logarithmic CO_2 RF law. This is a standard exponential lagged model for the greenhouse effect.

Supplementary Equation 19 was solved in closed form during each analysis period $t_{i-1} < t < t_i$, which is possible since the temperature is assumed to be constant during each period, using an improved technique described in the sections on the GMTS calculations in PAGE-ICE above.

The resulting difference between the cumulative RF from the nonlinear SAF and constant legacy SAF is plotted in Supplementary Figure 4 for the climate scenarios considered. The nonlinear SAF is marginally higher than the legacy SAF for the lower emissions scenarios, before dropping below the legacy SAF as the Arctic sea ice and land snow covers disappear (Figure 7 in the main article). As a result, the RF based on the nonlinear SAF is lower than the RF from the constant legacy SAF for the higher emissions scenarios; this effect is particularly strong for BAU in the 23rd century. This implies that the IAMs such as PAGE, DICE and FUND slightly underestimated the effect of the nonlinear SAF for the low emissions scenarios, and overestimated it for the medium and high emissions scenarios.

Supplementary Note 4

Our study is the first one to show that the strength of the PCF, measured by the resulting increase in GMST, declines after 2200 after the highest emission scenario considered (Figure 4 of the paper). This is due to the following factors:

- Decrease in the marginal effect of CO_2 emissions from the PCF on the RF and GMST as atmospheric CO_2 concentrations increase (Hope, 2006)
- Slowdown in the underlying GMST growth as global emissions stabilise, resulting in lower rates of permafrost thaw and the associated carbon emissions
- In some cases, exhaustion of permafrost carbon stock, which contributes to the drop in the annual CO_2 flux from permafrost beyond 2200
- Carbon removal from the atmosphere through CO_2 ocean uptake with time lag of several decades

Here we illustrate the last effect by plotting annual CO_2 fluxes from permafrost after the initial land uptake, the resulting additional annual CO_2 ocean uptake (negative by definition) driven by the cumulative permafrost emissions, as well as the sum of the two (Supplementary Figure 3). The fluxes were computed using the PAGE-ICE model run under the RCP4.5 (medium emissions) and RCP8.5 (high emissions) scenarios out to 2300. Supplementary Figure 3 shows the means of 10,000 Monte-Carlo experiments.

For RCP4.5, the annual permafrost flux starts to drop after 2100 when the GMST increase slows down, and the annual ocean uptake of the permafrost-related carbon remains nearly constant. As a result, the sum of the two fluxes, which is the net permafrost-related CO_2 flux contributing to the carbon pool that stays in the atmosphere indefinitely (on the timescales considered), declines quite rapidly in the 22nd century. This means that the long-term atmospheric CO_2 pool due to the permafrost emissions does not build as fast, therefore limiting the corresponding GMST effect of the PCF.

For RCP8.5, the permafrost flux experiences a drop after 2150, which is due to the GMST slowdown for this scenario, as well as to the permafrost stock exhaustion in the most extreme cases. Following this drop, the permafrost-driven CO_2 ocean uptake experiences only a relatively slow decline after 2200, resulting in an accelerated drop in the net CO_2 flux associated with the permafrost in the 23rd century. As for RCP4.5, this causes a slowdown in building up of the long-term atmospheric CO_2 pool associated with permafrost, and is one of the reasons why the strength of the GMST effect of the PCF decreases marginally after 2200 for the high emissions scenarios (see Figure 4 in the main article).

Supplementary Note 5

Recent PCF studies using LSMs and IAMs

Previous studies of the effects of the PCF relied on

(i) importing CO_2 and methane fluxes from SiBCASA simulations for land permafrost under SRES climate scenarios into PAGE09 IAM (Hope & Schaefer, 2016),
(ii) importing CO_2 and methane fluxes from the Schneider von Deimling permafrost ecosystem model (Schneider von Deimling et al., 2014) into DICE IAM (González-Eguino & Neumann, 2016),
(iii) using linear exponential lagged model for permafrost emissions in response to GMST increase (Schaefer et al., 2011) integrated dynamically in DICE IAM (Kessler, 2017), calibrated based on historic permafrost data (Hugelius et al., 2014),
(iv) coupling CO_2 land permafrost fluxes from the JULES and ORCHIDEE-MICT ecosystems models with the IMOGEN climate emulator of intermediate complexity run both under historic data and RCP climate scenarios (Burke et al., 2017; 2018).

Studies (i)-(iii) estimated climatic as well as economic impacts of the PCF, with (i) focusing on climate damages, and (ii) & (iii) on climate control. Neither (i) nor (ii) modelled the coupling effects of the PCF, and neither developed a dynamic emulator. Study (iii) introduced the coupling effects through a linear emulator, but the latter does not account for nonlinearities in the PCF, including the difference between the CO_2 and methane components, and its calibration did not rely on results from process-based ecosystems models as a reference point for future climate scenarios. While (iv) is the most complete study of the climatic impact of the PCF to date, it did not consider the methane component and stopped short of investigating the economic implications. Its proposed new policy metric, frozen carbon residence time (FCRt), appears to oversimplify the dynamics of land permafrost emissions on the global scale, being based on algebraic curve fitting for the remaining carbon pool and current emissions rate instead of a nonlinear dynamic model driven by the equilibrium cumulative carbon emitted, which is introduced in our work (see the Methods section of the paper). Furthermore, the IMOGEN climate emulator employed in these studies assumes that the CO_2 ocean carbon uptake is neutralized with remnant carbon emissions (Mauritsen & Pincus, 2017), which could have affected the estimates of the strength and timing of the PCF impacts. In this paper we use the latest multi-model assessment of the atmospheric CO_2 response function with two characteristic timescales for the ocean uptake (Joos et al., 2013).

Recent SAF studies using IAMs

The only previous study attempting to isolate the effects of the SAF employed DICE IAM to explore climatic and economic implications of the sea ice decline under the 2°C scenario (González-Eguino et al., 2017). The cumulative RF from the SAF (sea ice component only) was added exogenously as a piece-wise linear function of time until 2100, calibrated according to historic data (Flanner et al., 2011) and estimates for the cases of the September ice-free and year round ice-free Arctic (Holland et al., 2006; Hudson, 2011). This approach ignores the fact that a constant baseline level of the SAF is already included in the IAMs such as PAGE, DICE and FUND implicitly through the $2xCO_2$ equilibrium climate sensitivity (ECS) parameter, therefore leading to double-counting. The study did not consider other climate scenarios, particularly those with higher levels of warming that are characterised by nonlinear transitions in the SAF seen in climate model simulations, and did not introduce a dynamic emulator of the SAF that could be applied to multiple climate scenarios.

Supplementary Discussion

Comparison between the 2.5^{o}C target scenario and the 1.5^{o}C & 2°C scenarios

In its general form PAGE-ICE models two sets of emissions scenarios, climates and the associated economic effects simultaneously. This allows one to perform statistical significance tests for the difference between any given pair of emissions scenarios. Supplementary Figure 23 shows probability density functions of the differences between the total economic effects of climate change for following scenario pairs: 2.5^{o}C and 2°C targets, and 2.5^{o}C and 1.5^{o}C

targets. The results of the statistical comparison between the 2°C and 1.5°C target scenarios are presented in the Discussion section of the paper (see Figure 6 therein).

The probability distributions were computed separately for each pair of scenarios using 100,000 Monte-Carlo iterations in PAGE-ICE. Both 2°C and 1.5°C target scenarios use SSP1 as the underlying socio-economic pathway, while the 2.5°C scenario has elements of other SSPs according to the scenario definitions in the Methods, even though it is still dominated by SSP1. This means that the statistical comparisons between the economic effects of the 2.5°C scenario and the 2°C & 1.5°C scenarios have an additional dimension associated with using different GDP and population projections. Previous analysis with PAGE09 indicated that the choice of the underlying socio-economic pathway has strong influence on the estimates of the total economic effect of climate change, and in some cases could be more important than the underlying emissions pathway (Hope & Hope, 2013). In addition, the use of equity weighing based on per capita consumption requires careful ethical considerations when comparing between two distinct GDP and population pathways, since in this case having a smaller but richer population could lead to higher overall utility (Anthoff et al., 2009).

Based on the means of the distributions in Supplementary Figure 23, we conclude that the 2.5°C scenario is, on average, $218 trillion more expensive than the 2°C scenario and $227 trillion more expensive than the 1.5°C scenario when the nonlinear PCF and SAF representations are used. While the former result is significant at the 5% level, the latter is neither significant at the 5% nor at the 10% levels. This is due to the extensive negative tail associated with high mitigation cost of the 1.5°C scenario. However, the multiple arguments spelled out in the Discussion section suggest that it would be prudent to aim for emissions well below the 2°C target.

Supplementary References

1. Van Vuuren, D. P. et al., The representative concentration pathways: an overview, *Climatic Change*, 109(1-2), 5, 2011
2. Fernández-Martínez, M. et al., Global trends in carbon sinks and their relationships with CO2 and temperature, *Nature Climate Change*, 9, 73–79, 2019
3. Keenan, T. F., Recent pause in the growth rate of atmospheric CO2 due to enhanced terrestrial carbon uptake, *Nature Communications*, 7, 13428, 2016
4. Omta, A. W., Dutkiewicz, S., & Follows, M. J., Dependence of the ocean-atmosphere partitioning of carbon on temperature and alkalinity, *Global Biogeochemical Cycles*, 25(1), 2011
5. Eyring, V. et al., Long-term ozone changes and associated climate impacts in CMIP5 simulations, *Journal of Geophysical Research: Atmospheres*, 118(10), 5029-5060, 2013
6. Hansen, J. A. M. E. S., Sato, M., & Ruedy, R., Long-term changes of the diurnal temperature cycle: implications about mechanisms of global climate change, *Atmospheric Research*, 37(1-3), 175-209, 1995
7. Alexeev, V. A., Langen, P. L., & Bates, J. R., Polar amplification of surface warming on an aquaplanet in "ghost forcing" experiments without sea ice feedbacks, *Climate Dynamics*, 24(7-8), 655-666, 2005

8. McKinsey, Abatement Cost Curve; International Energy Agency World Energy Outlook, *US Environmental Protection Agency & European Environment Agency (EEA)*, 2009
9. Piontek, F. et al., Economic Growth Effects of Alternative Climate Change Impact Channels in Economic Modeling, *Environmental and Resource Economics*, pp1-29, 2018
10. Collins, M. et al., Long-term Climate Change: Projections, Commitments and Irreversibility, in: Climate Change 2013: The Physical Science Basis, Contribution of Working Group I to the Fifth Assessment Report of the Intergovernmental Panel on Climate Change, *Cambridge University Press*, 2013
11. Soden, B. J., Held, I. M., Colman, R., Shell, K. M., Kiehl, J. T., & Shields, C. A., Quantifying climate feedbacks using radiative kernels, *Journal of Climate*, 21(14), 3504-3520, 2008
12. Pistone, K., Eisenman, I., & Ramanathan, V., Observational determination of albedo decrease caused by vanishing Arctic sea ice, *Proceedings of the National Academy of Sciences*, 111(9), 3322-3326, 2014
13. Cao, Y., Liang, S., Chen, X., & He, T., Assessment of Sea Ice Albedo Radiative Forcing and Feedback over the Northern Hemisphere from 1982 to 2009 Using Satellite and Reanalysis Data, *Journal of Climate*, 28(3), 1248-1259, 2015
14. Schneider von Deimling, T. et al., Observation-based modelling of permafrost carbon fluxes with accounting for deep carbon deposits and thermokarst activity, *Biogeosciences Discussions*, 11(12), 16599-16643, 2014
15. Burke, E. J., Chadburn, S. E., Huntingford, C., & Jones, C. D., CO2 loss by permafrost thawing implies additional emissions reductions to limit warming to 1.5 or 2° C, *Environmental Research Letters*, 13(2), 024024, 2018
16. Mauritsen, T., & Pincus, R., Committed warming inferred from observations, *Nature Climate Change*, 7(9), 652, 2017
17. Holland, M. M., Bitz, C. M., & Tremblay, B., Future abrupt reductions in the summer Arctic sea ice, *Geophysical Research Letters*, 33(23), 2006
18. Hudson, S. R., Estimating the global radiative impact of the sea ice–albedo feedback in the Arctic, *Journal of Geophysical Research: Atmospheres*, 116(D16), 2011
19. Hope, C. & Hope, M., The social cost of CO 2 in a low-growth world, *Nature Climate Change*, 3(8), 722, 2013

The Pentland Centre for Sustainability in Business
Lancaster University
Lancaster
United Kingdom
LA1 4YX

T +44 1524 510694
E pentlandcentre@lancaster.ac.uk
W www.lancaster.ac.uk/pentland
@PentlandCentre

Connecting great minds in science to great minds in business
