# Supplementary material for: Climate policy implications of nonlinear decline of Arctic land permafrost and other cryosphere elements
Source: Nat Commun. 2019 Apr 23;10:1900. doi: 10.1038/s41467-019-09863-x (PMC6478735; doi:10.1038/s41467-019-09863-x)
Supplement: Supplementary file 3 — Description of Additional Supplementary Files [file 41467_2019_9863_MOESM3_ESM.pdf]

Description of Additional Supplementary Information

Yumashev et al., Nature Communications

Codes

File Name: **Supplementary Code 1**
Description:

Default version of the PAGE-ICE model (v6.22) used in the study. The Excel workbook includes extensive references to the underlying datasets, and a Readme tab with a brief description of the model and how to run it. See Supplementary Note 1 for further information. PAGE-ICE requires @RISK add-in by Palisade to run statistical (Monte-Carlo) simulations in Excel. Free trial version of @RISK is available at https://www.palisade.com/trials.asp

An open source Julia version of PAGE-ICE will be made available on the Mimi platform shortly. For further details see https://www.mimiframework.org/

File Name: **Supplementary Code 2**
Description:

MATLAB scripts for calibrating the PCF emulator using SiBCASA and JULES simulations.

File Name: **Supplementary Code 3**
Description:

Scripts for calibrating the SAF emulator using CMIP5 models and ALL/CLR method.

First component: modified Winton's ALL/CLR script in Python to calculate the RF due to the SAF from a given climate model (ACCESS1-3 in the example provided). Includes the following extensions: multiple CMIP5 models (separate scripts), transient simulations from pre-industrial until 2300 under RCP8.5, pre-industrial sea ice and land snow domains, fixed pre-industrial clouds experiment, comprehensive checks.

Second component: MATLAB script for calculating the SAF (3 components, with either dynamic or fixed clouds) and analysing the multi-model CMIP5 statistics using the RF time series produced by the modified Winton script.

File Name: **Supplementary Code 4**
Description:

Python scripts for calculating population-weighted absolute temperatures in the PAGE regions for the 1979-2005 climatology using Era-Interim reanalysis and gridded population data from SEDAC.

File Name: **Supplementary Code 5**
Description:

Python script for calculating amplification factors for the PAGE regions based on the complete set of CMIP5 RCP simulations between 2006 and 2100.

Datasets

File Name: **Supplementary Data 1**
Description:

Statistical simulations of the default PAGE-ICE model (v6.22), with and without the nonlinear PCF and SAF corrections under multiple scenarios, which are presented in the study.

File Name: **Supplementary Data 2**
Description:

Simulations of permafrost carbon emissions from SiBCASA and JULES LSMs.

First component: permafrost CO_2 and methane simulations using SiBCASA LSM with 5 CMIP5 models under 2 climate scenarios, along with the underlying GMST and permafrost temperature simulations. Referred to as “Perm_simulations_SiBCASA.xlsx” in the code.

Second component: permafrost CO_2 simulations using JULES LSM with 22 CMIP3 models under 3 climate scenarios, along with the underlying GMST and permafrost temperature simulations. The version of JULES used here includes vertically scaled respiration in thawed permafrost soils. Referred to as “Perm_simulations_JULESdR.xlsx” in the code.

File Name: **Supplementary Data 3**
Description:

Calibration results for the PCF emulator of SiBCASA and JULES simulations.

File Name: **Supplementary Data 4**
Description:

RF due to the SAF from 16 CMIP5 models calculated using the modified Winton's ALL/CLR script. The results are presented as time series from 1865 until 2285, separately for the Arctic sea ice, land snow and rest of the world SAF components, and both for the dynamic and fixed clouds. Referred to as "CMIP5_models_fsaf_30year_running_mean_dynamic_and_fixed_clouds.xlsx" in the code.

File Name: **Supplementary Data 5**
Description:

Additional datasets for calibrating PAGE-ICE v6.22, including:

- Country-level definitions of the PAGE model regions.
- Historic GMST time series to calculate the base year climatological temperatures in PAGE-ICE.
- Base year cumulative CO2 emissions, annual GHG emissions and GDP PPP for the PAGE model regions.
- Reconstruction of the CO2 cycle response function from Joos et al. (2013), with re-calibration of the model with 3-exponentials.
- Reconstruction of the economic impact function from Burke et al. (2015).
- Estimates for the ratio between the methane and CO2 components of permafrost carbon emissions based on observational data (Schädel et al., 2016). Required for quantifying methane emissions in the JULES simulations.
- Amplification factors for the PAGE regions based on the complete set of CMIP5 RCP simulations between 2006 and 2100.
- Population-weighted absolute temperatures in the PAGE regions for the 1979-2005 climatology based on Era-Interim reanalysis and gridded population data from SEDAC.

Plots

File Name: **Source Data**
Description:

Raw data underlying the results reported in the plots.
